# Crystallographic and Geometrical Dependence of Water Oxidation Activity in Co-Based Layered Hydroxides

**DOI:** 10.1021/acscatal.3c01432

**Published:** 2023-07-24

**Authors:** Roger Sanchis-Gual, Diego Hunt, Camilo Jaramillo-Hernández, Alvaro Seijas-Da Silva, Martín Mizrahi, Carlo Marini, Víctor Oestreicher, Gonzalo Abellán

**Affiliations:** † Instituto de Ciencia Molecular (ICMol), Universidad de Valencia, Catedrático José Beltrán 2, 46980 Paterna, Valencia, Spain; ‡ Departamento de Física de la Materia Condensada, GIyA. Instituto de Nanociencia y Nanotecnología, CNEA-CAC-CONICET, Av. Gral. Paz, 1650 San Martín, Buenos Aires, Argentina; § Instituto de Investigaciones Fisicoquímicas Teóricas y Aplicadas (INIFTA), Departamento de Química, Facultad de Ciencias Exactas. Universidad Nacional de La Plata, CCT La Plata- CONICET, Diagonal 113 y 64, 1900 La Plata, Argentina; ∥ Facultad de Ingeniería, Universidad Nacional de La Plata, Calle 1 esq. 47, 1900 La Plata, Argentina; ⊥ CELLS−ALBA Synchrotron, Cerdanyola del Vallès, 08290 Barcelona, Spain

**Keywords:** layered materials, layered hydroxides, electrocatalysis, water splitting, oxygen evolution reaction, Co-based electrocatalyst, DFT calculations

## Abstract

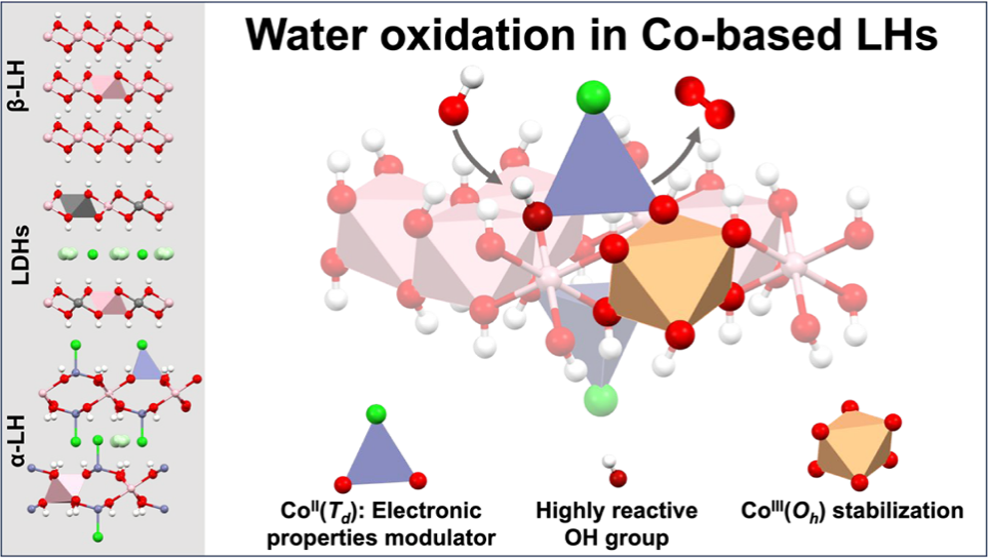

Cobalt-based layered hydroxides (LHs) stand out as one of the best families of electroactive materials for the alkaline oxygen evolution reaction (OER). However, fundamental aspects such as the influence of the crystalline structure and its connection with the geometry of the catalytic sites remain poorly understood. Thus, to address this topic, we have conducted a thorough experimental and in silico study on the most important divalent Co-based LHs (i.e., α-LH, β-LH, and LDH), which allows us to understand the role of the layered structure and coordination environment of divalent Co atoms on the OER performance. The α-LH, containing both octahedral and tetrahedral sites, behaves as the best OER catalyst in comparison to the other phases, pointing out the role of the chemical nature of the crystalline structure. Indeed, density functional theory (DFT) calculations confirm the experimental results, which can be explained in terms of the more favorable reconstruction into an active Co(III)-based oxyhydroxide-like phase (dehydrogenation process) as well as the significantly lower calculated overpotential across the OER mechanism for the α-LH structure (exhibiting lower Egap). Furthermore, ex situ X-ray diffraction and absorption spectroscopy reveal the permanent transformation of the α-LH phase into a highly reactive oxyhydroxide-like stable structure under ambient conditions. Hence, our findings highlight the key role of tetrahedral sites on the electronic properties of the LH structure as well as their inherent reactivity toward OER catalysis, paving the way for the rational design of more efficient and low-maintenance electrocatalysts.

## Introduction

In the research of efficient renewable energies, hydrogen appears as the fuel of the future.^1−3^ Currently, the most promising green alternatives are based on its generation through renewable energy-based electrolysis. However, in the water-splitting process, the oxygen evolution reaction (OER) remains as the bottleneck of the entire process, suffering slow kinetics (>10 000 lower than hydrogen evolution reaction), therefore requiring high overpotentials, which account for the majority of energy losses.^4^

Since the first report of a layered hydroxide (LH) for electrochemical water splitting,^5^ enormous progress has been achieved to improve the electrochemical performance of these earth-abundant two-dimensional compounds which compete favorably with the benchmark based on precious metal oxides such as Ir and Ru. Along this front, several parameters have been investigated: the influence of different morphologies,^6−8^ interlayer spaces,^9^ metallic compositions,^10−12^ clustering,^13^ and even structural instability of the layers during the water-splitting activity.^14^ In general, the catalytic enhancements are usually a consequence of the increment of the electrochemical surface areas, the capability to adsorb OH^–^ ions, the diffusion properties, and/or the intrinsic activities of electroactive sites.^15^ However, a fundamental parameter such as the case of the specific role of the crystalline LH structure, as well as the coordination environment of the cation (i.e., geometrical-site dependency), has been surprisingly set aside in the literature, when even nowadays the role of octahedral^16−22^ and tetrahedral^23−28^ electrocatalytic OER centers is still a matter of discussion in the case of earth-abundant oxide and spinel phases.

Hence, in this work, we decide to perform an exhaustive exploration of the role of cationic coordination of LHs on the OER performance for the first time. For this, we have selected the widely studied divalent cobalt-based hydroxides as a paradigmatic example of highly electroactive phases showing rich structural diversity. Certainly, we have synthesized and fully characterized a complete Co-based LH family in the form of nonexpanded β-LH (brucite-like structure), as well as expanded phases such as LDH (hydrotalcite-like structure) and α-LH (simonkolleite-like structure), providing unmistakable clear fingerprints for their straightforward identification, as they have traditionally been widely confused in the literature. It is important to highlight that, since our study aims to understand the specific role of the crystal structure, divalent cobalt and nonelectroactive trivalent cations have been employed, exclusively. At first glance, the electrochemical characterization confirms that Co-based phases containing interlayer anions in their structures (α-LH and LDH) behave as better electrocatalytic materials in comparison to the β-LH, as expected for a nonexpanded structure with lower electrolyte diffusion. Nevertheless, the electrochemical analysis confirms the superior catalytic behavior of the α-LH (containing tetrahedral Co sites) in comparison to the LDH. Specifically, for the α-LH structure, the onset potential is reduced and the kinetics of the reaction, estimated by the Tafel slope and TOF values, are greatly enhanced. We discussed the underlying mechanism with a wide range of spectroscopic, diffraction, and theoretical methods (DFT + U), followed by electrochemical water oxidation assessments, showing that the presence of tetrahedral environments induces drastic changes in the electronic properties (markedly reducing the Egap), favors the massive and permanent reconstruction of the α-LH into a highly Co^III^-based reactive oxyhydroxide-like phase (dehydrogenation process), and presents the lower computed overpotential values across the OER mechanism. Indeed, by an ex situ structural and spectroscopic analysis, we have demonstrated that α-LH transforms into an oxyhydroxide-like phase stable under ambient conditions for days. Thus, this work introduces a comprehensive study of the crystallographic nature of LH phases and their role in the OER performance, pointing out α-LH structures as a promising phase for the incorporation of electroactive trivalent cations keeping in mind the rational design of low-cost highly efficient electrocatalytic materials.

## Results and Discussion

In order to systematically study and rationalize the influence of the crystallographic structure of Co-based layered hydroxides (LHs) on the water oxidation performance, the following typical LH phases have been prepared by direct synthesis: brucite-like (β-LH), hydrotalcite-like (layered double hydroxide, LDH), and simonkolleite-like (α-LH) structures.

Specifically, β-LH phases are nonexpanded structures, exhibiting basal space distances (*d*_BS_) lower than 5 Å, and containing divalent cations exclusively adopting octahedral environments, M^II^(*O*_h_).^29^ On the other hand, layered double hydroxide (LDH) phases—which represent the most famous member of the LHs’ family reported so far^30^—consist of positively charged layers composed of both divalent and trivalent cations located in octahedral environments, M^II^(*O*_h_) and M^III^(*O*_h_), respectively, where the charge excess is compensated by the incorporation of interlaminar anions, which expands the *d*_BS_ to values greater than 7 Å. In this sense, different LDH structures represented by the chemical formula *M*_1–*x*_^II^*M*_*x*_^III^ (OH)_2_*A*_*x*/*n*_^*n*–^·*m*(H_2_O) can be obtained by purely electrostatic anion-exchange reactions, leading to a plethora of interesting materials for applications in anion-exchange, drug-delivery, (electro)catalysis, or (opto)magnetic devices, to name a few.^31−33^ It is worth mentioning that since the idea of this work is to perform an investigation of the crystal structure itself, only divalent Co electroactive centers will be considered. Thus, an LDH phase containing nonelectroactive cations such as Al^III^(*O*_h_) has been selected.

Finally, α-LH phases are also expanded structures containing anions in the interlayer space, the reason why in some cases they are also known as basic salts.^34^ The specific crystallographic structure of the α-LHs strongly depends on the divalent cations, which can adopt either octahedral (*O*_h_) or tetrahedral (*T*_d_) environments.^29,35,36^ In the particular case of Co-based α-LHs, the phase consists of a simonkolleite-like structure,^37^ containing both *O*_h_ and *T*_d_ crystallographic environments, and covalent interactions between the anion and the Co^II^(*T*_d_) sites, obeying the formula Co_1–*x*_^*O*_h_^Co_*x*_^*T*_d_^ (OH)_2_*A*_*x*/*n*_^*n*–^ · *m*(H_2_O).^38,39^

**Scheme 1:**
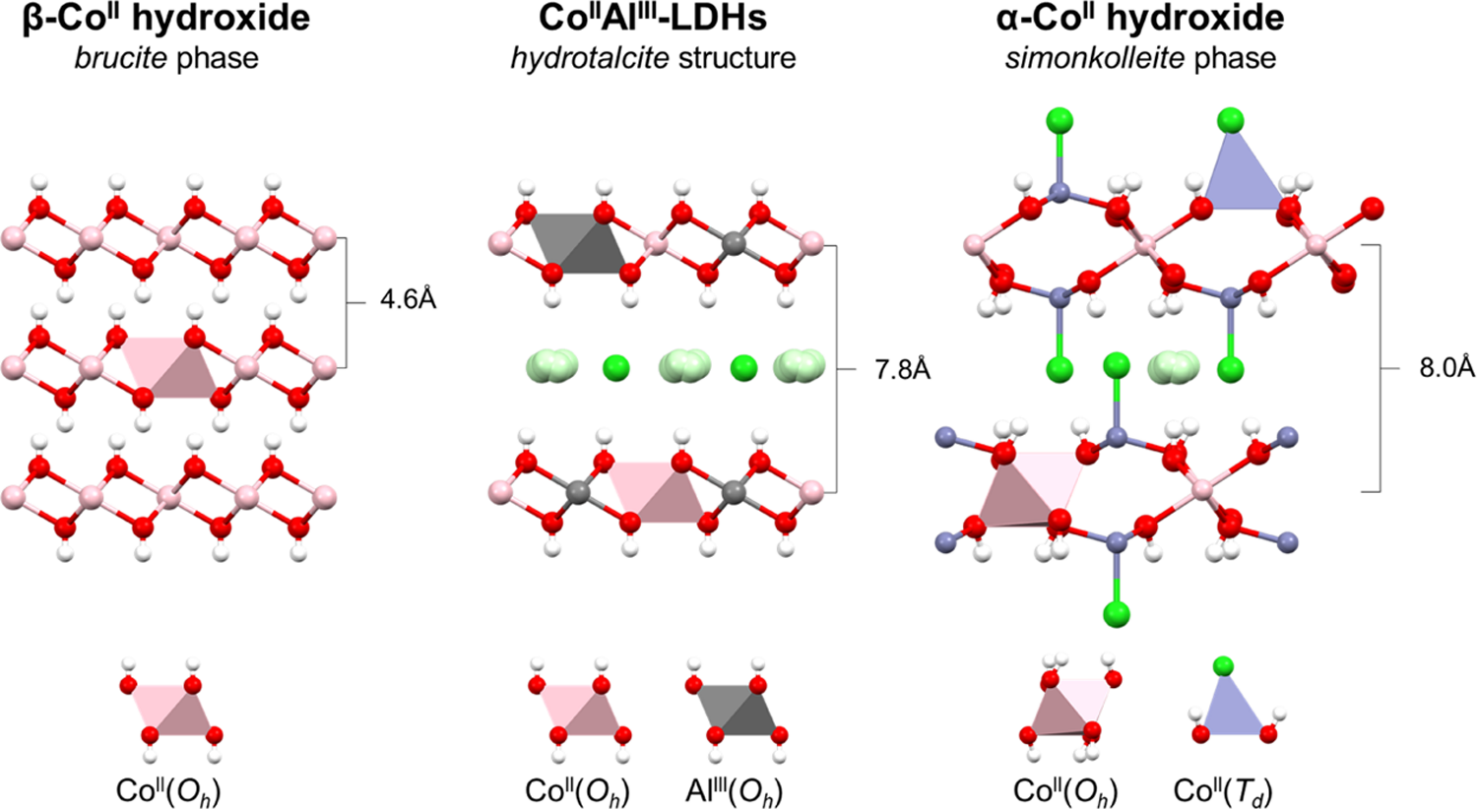
Schematic Representation of the Different Crystallographic Structures for Co-Based Layered Hydroxides Studied in This Work, Highlighting Their Characteristic Crystallographic Environments (Octahedral—*O*_h_—and Tetrahedral—*T*_d_—Motifs) and the Reported Basal Space Distances (*d*_BS_)

To sum up, while β-LH is composed of divalent cations located in octahedral environments, LDH contains divalent and trivalent cations in the same octahedral positions, and α-LH exhibits divalent metal in both octahedral and tetrahedral environments. The comparison between these structures represents a perfect scenario for deciphering the crystallographic and geometrical dependence of water oxidation activity in Co-based layered hydroxides. The schematic representation of each Co-based LH structure is depicted in Scheme 1.

**Figure 1:**
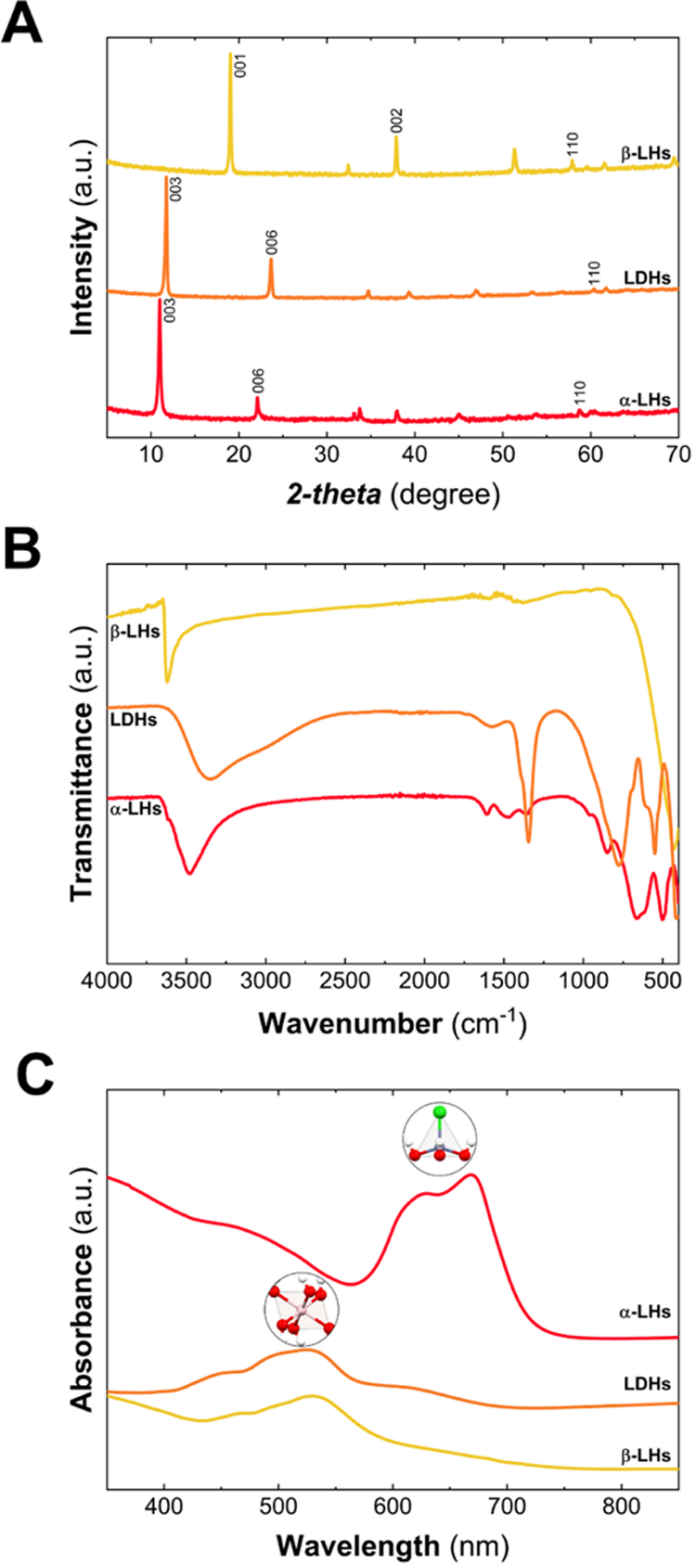
Structural characterization of Co-based LHs. PXRD patterns highlight the layered nature of the structures as denoted by the *00l* reflections, while the intralayer distance is denoted by the 110 reflection (A). Attenuated total reflectance–Fourier transform infrared (ATR–FTIR) spectra depict a clear change in the water-related bands, highlighting the presence of intralayer water (B). UV–vis spectra point out the differences in the cobalt octahedral environment as well the presence of tetrahedral Co(II) in the case of α-LH (C).

Figure 1 depicts the structural characterization recorded over the obtained phases through well-established synthetic protocols leading to similar micrometric hexagonal crystals, as depicted in Figure S1 (see “Co-based LH synthesis”, Experimental section in the Supporting Information).

The powder X-ray diffraction (PXRD) patterns shown in Figure 1A confirm the layered nature of the phases as it can be inferred by the presence of the reflections at low 2θ values, indexed as 00*l*. The obtained interlayer space (*d*_BS_) values are 4.66, 7.52, and 8.04 Å for β-LH, LDH, and α-LH phases, respectively. The estimation of the intralayer (*a*) parameters can be extracted from the 110 reflections at higher 2θ values, corresponding to ca 3.18, 3.06, and 3.14 for β-LH, LDH, and α-LH phases, respectively. As expected, the lower *a* value for the LDH is based on the incorporation of a smaller cation such as Al^III^(*O*_h_) within the layers (Co_*O*_h__^II^ = 0.745 Å; Al_*O*_h__^III^ = 0.535 Å).^40,41^Table S1 compiles the cell parameters as a function of LH phases, which are in good agreement with previous reports.^29,35,42^

Attenuated total reflectance–Fourier transform infrared spectroscopy (ATR–FTIR) also provides valuable structural information about each LH phase, as depicted in Figure 1B. The β-LH phase can be distinguished by the presence of an intense and sharp band at around 3630 cm^–1^ attributed to the O–H stretching mode, characteristic of free-OH groups in brucite-like structures.^29^ In the case of the expanded structures, the presence of interlayer water molecules can be confirmed by the broadband centered at ca 3400 cm^–1^ (O–H stretching mode) and an extra peak at around 1600 cm^–1^ (H_2_O bending mode). The sharper and more defined band observed in the case of the α-LH phase is attributed to a higher water confinement degree.^41^ In the particular case of the LDH structure, the bands at 1345 and 800 cm^–1^ confirm the incorporation of carbonate molecules, which was to be expected considering that the synthetic method uses urea as an alkalinization agent.^42^ Finally, signals below 1000 cm^–1^ are associated with Co–O stretching and Co–OH bending vibrations. Interestingly, these vibrations strongly depend on LHs phase’s identity, resulting in a fingerprint to easily distinguish the LH structures (see Table S2, SI).

UV–vis diffuse reflectance spectroscopy is a suitable technique in LH characterization, as it can provide information on both metallic coordination environments and oxidation states, in contrast to conventional X-ray photoelectron spectroscopy (XPS) measurements, as we recently reported for Co-based LHs.^43^ As in the case of metal transition complexes, the UV–vis region provides information about the ligand field excitation associated with the oxidation state and coordination environment of metallic centers, as well as the nature of the ligands.^44−46^Figure 1C depicts the spectra where the bands are related to metal-to-metal adsorption, i.e., *d–d* electronic transitions. The broad bands located around 500 nm are assigned to the ^4^T_1g_ to ^4^T_1g_(P) and ^4^A_2g_(F) transitions of Co^II^(*O*_h_), while the intense band with twin peaks at 629 and 668 nm, belonging to the ^4^A_2_(F) to ^4^T_1_(P) transition, corresponds to Co^II^(*T*_d_), which strongly depends on the coordinated anion’s identity.^35,38,47,48^ It is worth remarking that the bands ascribable to Co^II^(*O*_h_) environments evidence slight differences for each LH phase, which can be used as both (i) LHs’ fingerprint and, additionally, (ii) to evidence changes in the electronic structure (see Figure S2 and Table S3, SI).

**Figure 2:**
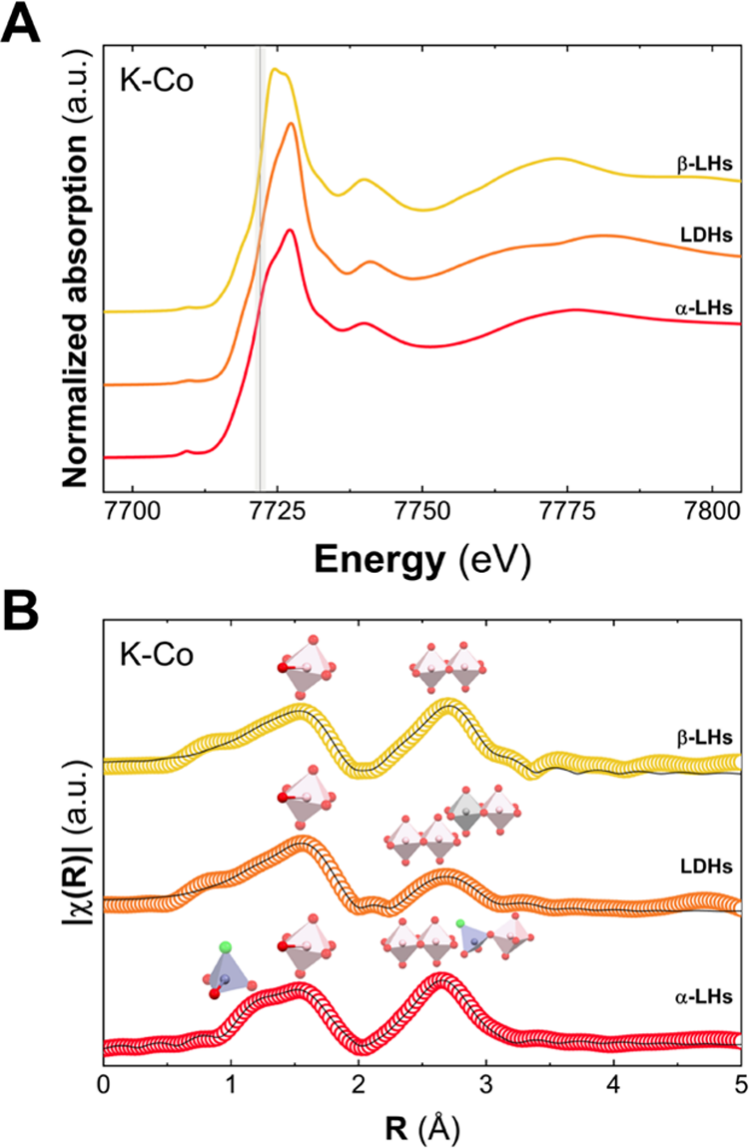
Normalized XANES spectra at the Co K-edge. The black line depicts the expected position for cobalt atoms exhibiting oxidation state +2 (A). Fourier transform of the extracted κ^2^-weighted EXAFS oscillations, for the measured samples—circles—and their corresponding fittings—black line. First peaks are attributed at Co–O distances (pink octahedron). In the case of α-LHs, the shorter value is assigned to tetrahedral Co–O distances (blue tetrahedron). Second peaks consider the Co–Co distances in the case of β-LHs (pink–pink polyhedrons) and α-LHs (pink–pink and pink–blue polyhedrons) and Co–Co (pink–pink polyhedrons) and Co–Al (pink–gray polyhedrons) ones for LDH (B). In all of the cases, the XAS spectra are represented without phase correction.

In order to further characterize this Co-based LH family, X-ray absorption spectroscopy (XAS) measurements were performed in the CLÆSS BL22 beamline at ALBA Synchrotron. Figure 2A depicts the X-ray absorption near-edge structure (XANES) spectra for the Co–K-edge. In all of the samples, the presence of Co^II^ can be successfully confirmed since nondifferences between the position of the absorption edges (in gray) are observed, as expected.^35,49,50^

To delve into the structural features of each LH member, the extended X-ray absorption fine structure (EXAFS) regions have been also analyzed. Figure 2B depicts the Fourier transform (FT) of the EXAFS oscillations at the Co K-edge. For all of the samples, the two major contributions, located in the range 1–3 Å, represent the average distances (without phase correction) to the first and second coordination shell around the metallic atoms (i.e., Co–O and Co–Co/Al distances, respectively). Considering the first peak, a unique contribution characteristic for *O*_h_ sites is observed, except in the case of the α-LH sample where the presence of *O*_h_ and *T*_d_ is noticeable, as previously demonstrated by UV–vis measurements. In the case of the second peak, a marked decrease in the amplitude for the LDH sample is observed due to the role of Al cations, whose backscattered waves are practically out of phase, leading to destructive interferences. EXAFS fittings were also performed to obtain the structural parameters such as coordination numbers (*N*), interatomic distances (*R*), and structural disorder (σ^2^). The proposed models consider the following initial conditions: (i) a single Co–O distance for *O*_h_ cations, except in the case of α-LH where two Co–O coordination spheres (*O*_h_ and *T*_d_) with similar distances were employed,^35^ and (ii) a shell of M (Co and Al according to each sample) as second neighbors. The high quality of the fittings confirms a good match with the proposed models for each LH sample, reproducing pseudo-radial distributions. Distances to first and second neighbors are in good agreement with the crystallographic data and previous studies.^35,39^ Specifically, Co^II^(*T*_d_)–O = 1.9 Å, Co^II^(*O*_h_)–O = 2.1 Å, and Co^II^–Co^II^ = 3.1 Å (for more details. see Table S4, SI). As expected, in the case of β-LH and LDH phases, the average coordination number is ca 6, while in α-LHs structure, the presence of *T*_d_ sites reduces this value up to 5.

Hence, the establishment of Co-based LHs exhibiting nonexpanded and expanded structures (i.e., β-LH vs LDH & α-LH, respectively) and containing exclusively Co^II^ in octahedral and tetrahedral (i.e., β-LH & LDH vs α-LH) environments can be safely concluded.

**Figure 3:**
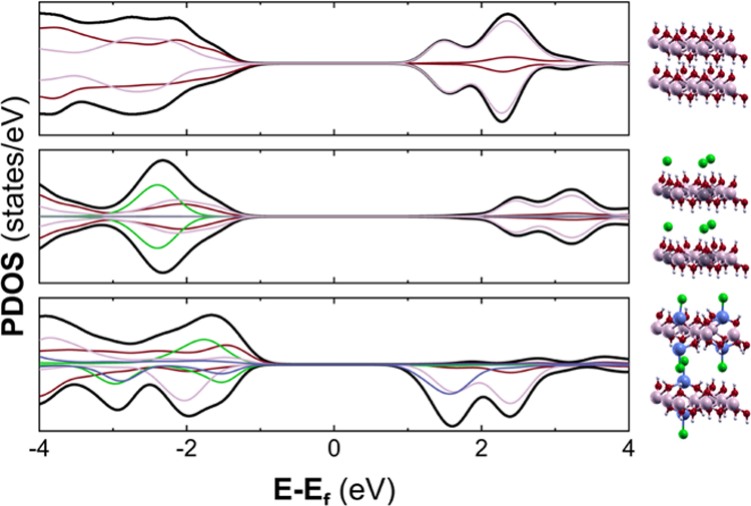
Total and projected density of states (PDOS) for Co-based LHs: β-LH (upper panel), LDH (middle panel), and α-LH (lower panel). Atoms are labeled according to Scheme 1: H—white, O—red, Al—gray, Cl—green, Co^II^(*O*_h_)—pink, and Co^II^(*T*_d_)—blue.

To gain further information on the electronic properties of these Co-based LH structures, an atomistic description based on density functional theory with Hubbard’s correction (DFT + U) has been carried out. Figure 3 depicts the projected and total density of states (PDOS) for the structures, where an electronic insulator behavior for all of these layered compounds is clearly seen from the total density of states. Nevertheless, a careful inspection of the PDOS demonstrates that both β-LH and LDH compounds present band gaps that exceed 2 eV, while the α-LH phase exhibits a considerable reduction in this value below 1.3 eV. This result is based on the large contribution of p states for covalently attached Cl atoms to the valence bands, while conduction bands are composed of p states from O atoms and *d* orbitals from Co centers (*O*_h_ and *T*_d_).^51^ Thus, unlike the purely electrostatic anion-layer interaction occurring in LDH compounds, the covalent interaction between anions and tetrahedral Co atoms remarkably modifies the electronic properties of α-LH compounds, making possible a more efficient charge transfer mechanism. As demonstrated in our previous works, the presence of these Co^II^(*T*_d_)-X motifs allows the manipulation of the electronic properties.^47,48,51^

Once the LH phases have been structurally and electronically characterized, we proceed with the analysis of their electrochemical performance in terms of the OER by measuring the water oxidation in a three-electrode cell in alkaline media (1 M KOH aqueous solution). For the sake of clarity, glassy carbon has been employed to avoid hidden catalyst–electrode interactions.^13^

**Figure 4:**
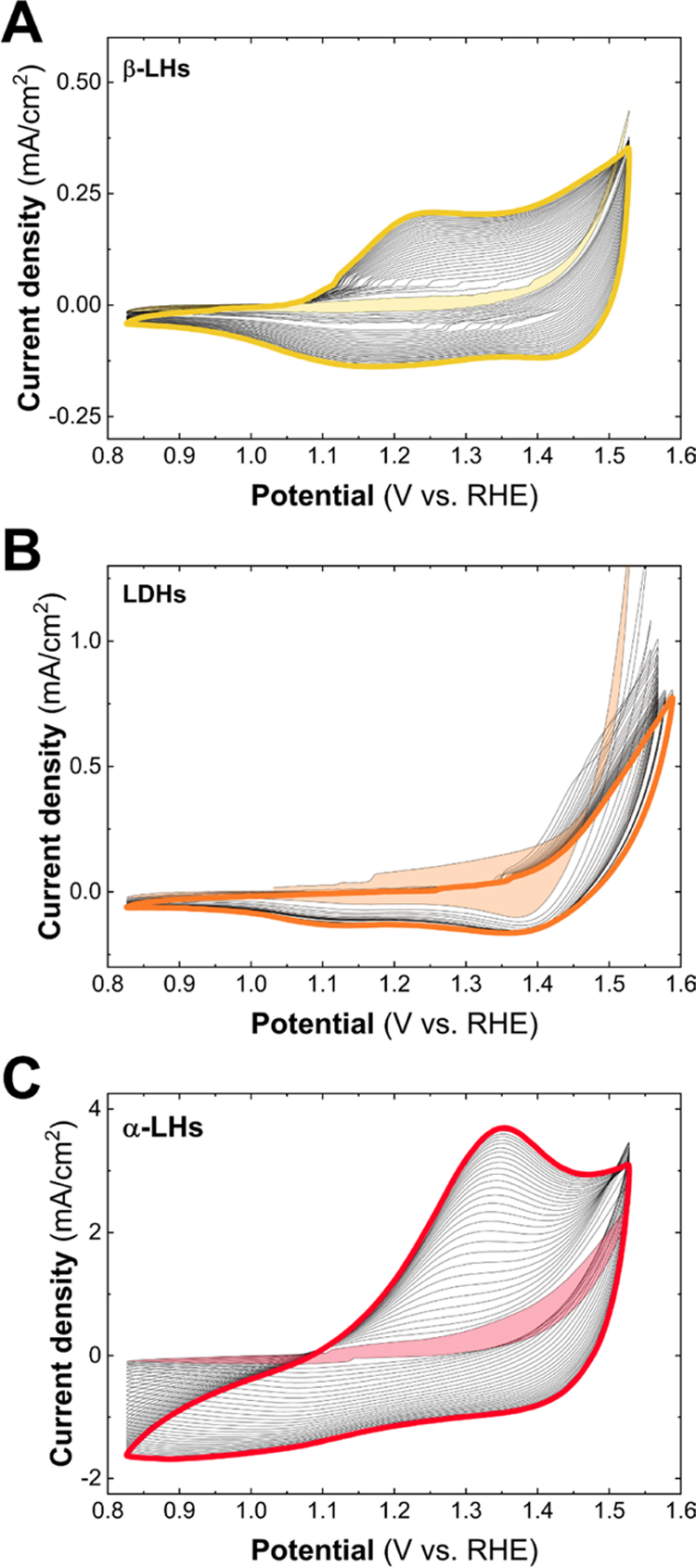
Activation processes carried out before the OER experiments consisted of 30 cyclic voltammetry curves performed in a 1 M KOH aqueous solution at 50 mV/s for each Co-based LH structure: β-LH (A), LDH (B), and α-LH (C). The first cycles are depicted as shading curves, and the final ones are presented by thick colored lines.

As a first step, cyclic voltammetry (CV) measurements were performed in order to drive the activation of the electroactive centers. As it is possible to observe in Figure 4, the peaks ascribable to the Co redox processes display a characteristic shape depending on their local environments (chemical identity), as well as a specific continuous increment during the successive cycles.^52^ The differences in the position of the redox peaks represent the first indication that the Co^II^ centers of the each LH structure are electrochemically different. Figure S3 depicts the cathodic charge, calculated as the integral of each CV curve, as a function of the activation cycle for all of the samples, giving a quantitative estimation of the whole activation process. In the cases of both β-LH and LDH phases, a considerable enhancement in these values up to 450% can be observed. Noteworthily, the α-LH phase shows an extraordinarily huge activation process, where the cathodic charge rises by more than 2000% in comparison to its initial values. The difference between α- and β-LH phases can be undoubtedly attributed to the *d*_BS_ values which control the accessibility of the electrolyte to the electroactive sites,^9^ i.e., expanded vs nonexpanded structures. However, it is quite surprising to record such different activations for α-LH and LDH structures since *d*_BS_ values are almost identical, emphasizing the role of the crystallographic structure.

**Figure 5:**
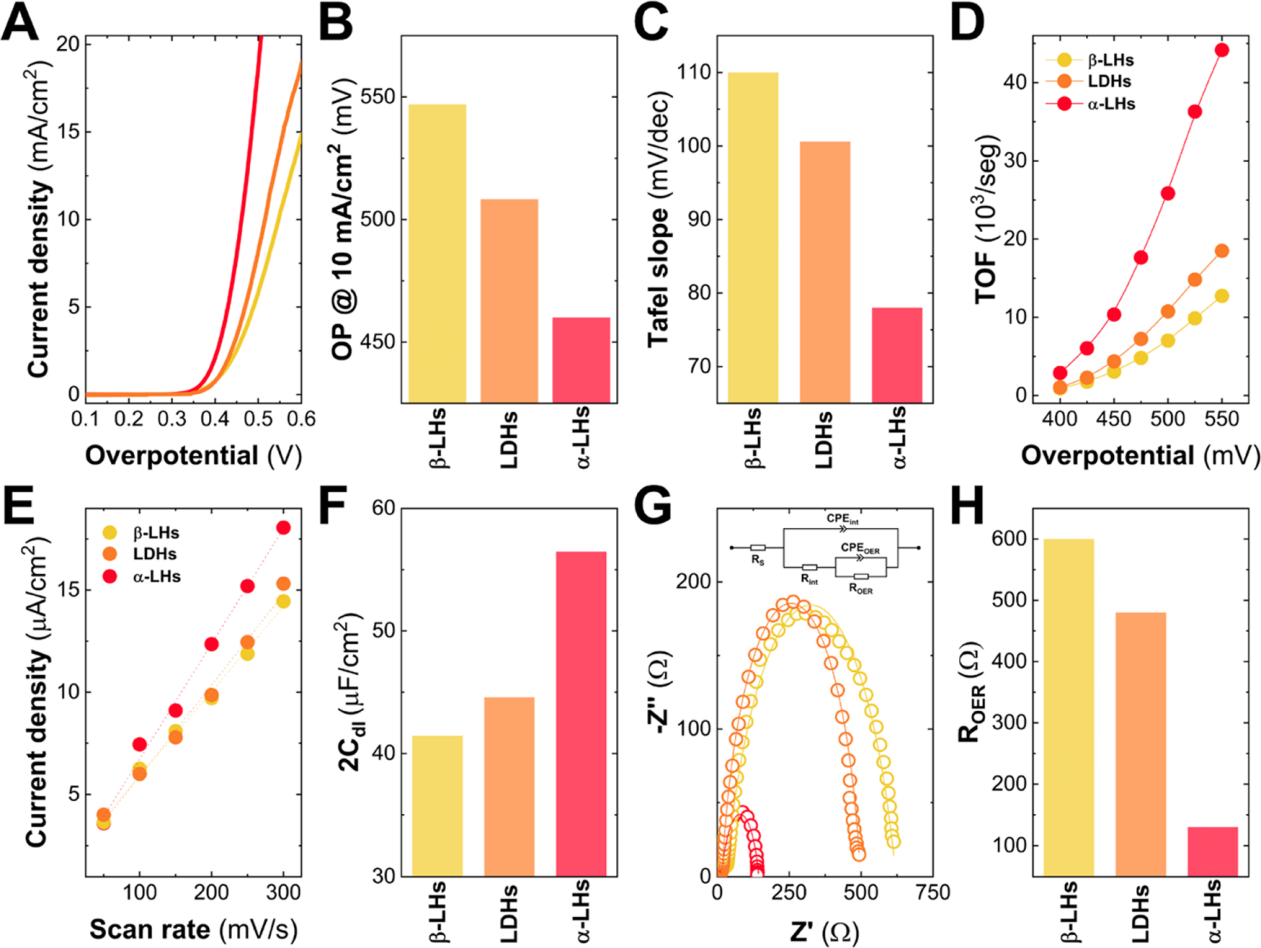
Electrochemical characterization for each Co-based LHs structure was recorded on a glassy carbon electrode collector. Linear sweep voltammetry curves were measured at 5 mV/s in 1 M KOH aqueous solution (A). Overpotential values required for a current density of 10 mA/cm^2^ (B). Tafel slopes were calculated from LSV data (C). Turnover frequency values obtained as a function of overpotential (D). Linear slopes representing the ECSA calculated from CVs performed in a non-faradaic region at different scan rates (E). ECSA values of the different structures (F). Nyquist plots of the different samples recorded at an overpotential of 0.4 V. Points correspond to experimental data, and lines are curves fitted with the equivalent circuit. Inset: equivalent circuit used (G). Resistance values of the process associated with the OER (H). These resistance values were calculated from the equivalent circuit (G).

Keeping this in mind, to provide further insights related to the electrochemical behavior of Co-based LHs, the electrocatalytic activity of these compounds was tested and compared by measuring linear sweep voltammetry (LSV) in 1 M KOH solution with a purity of 99.98%. Figure 5A depicts the curves under OER conditions by measuring the overpotential up to 0.6 V at a slow scan rate (5 mV/seg) to minimize the presence of capacitive currents.^53^ It is important to remark that these curves were not corrected with the solution resistance (*iR*, with *R* = 2 ± 1 Ω) to make a fair comparison of the intrinsic electrocatalytic behavior of each sample. To properly analyze the OER performance, key parameters such as (i) the overpotential (OP) required to get 10 mA/cm^2^, (ii) the Tafel slopes, and (iii) the turnover frequency (TOF) values are compiled in Figure 5B–D and Table S5. Regarding OP values, the α-LH phase arises as the best electroactive material, owing OP values almost 100 mV lower than their analogous compounds, β-LH and LDH phases. Moreover, in both Tafel slope and TOF values, calculated at the overpotential range of 400–525 mV, the α-LH sample presents the best figure of merits in comparison with their analogues. As in the activation process, these electrochemical differences between nonexpanded and expanded structures are evident. However, in the case of the expanded structures (i.e., α-LH & LDH) the results suggest that not only *d*_BS_ values are controlling the OER activity, highlighting changes in the catalytic activity of the α-LH sample.^54,55^

To verify the increment in the number of electroactive centers, the electrochemical surface area (ECSA) of each sample has been estimated. Specifically, ECSA was analyzed by obtaining the double-layer capacitance (Figure 5E,F). These values were extracted from the slopes of the fitted plots which correspond to the double-layer capacitance. On the one hand, expanded structures (LDH & α-LH) have higher ECSA values than the β-LH sample, as expected.^6,9,56^ Specifically, the α-LH phase presents values more than 25% larger than the LDH phase, which cannot be related to the slight difference in the basal spaces, thus pinpointing that the crystallographic structure can also increase considerably the number of electroactive species able to participate in the OER.

Once the electrochemical properties have been introduced, the kinetic features can be addressed. In this line, electrochemical impedance spectroscopy (EIS) measurements were also carried out. The obtained curves and the equivalent circuits used to fit the EIS data are presented in Figure 5G. Constant phase elements (CPEs), which are nonideal capacitances, were introduced to provide a good match with the experimental data because of the possible surface roughness, physical nonuniformity, and nonuniform distribution of the electroactive sites. The equivalent circuit is composed of a resistance representing the ionic transport through the solution and the current collectors (*R*_S_) connected in series with a first parallel branch (*R*_int_ and CPE_int_) corresponding to the interfacial contact between the material and the glassy carbon electrode. These three elements are observed in the high-frequency region. Nevertheless, in the low-frequency region, OER processes occurring on the surface are represented by a second parallel branch (*R*_OER_ and CPE_OER_). As can be observed from Figure 5H, once again the best values (i.e., the lowest resistance) are recorded for the α-LHs structure. The 6-fold reduction of *R*_OER_ can be associated with a larger number of active centers, as well as a higher intrinsic conductivity, which facilitates oxygen formation (thus increasing the OER performance).^57,58^ Therefore, in the case of the α-LH structure, the highest number of active sites and the decrease of the resistance connected with the OER process should be mainly related to the lower bandgap of this structure (as previously presented in Figure 3). This effect, which is governed by ligand-to-metal charge transfer, halide-to-Co^II^(*T*_d_),^47^ would result in the improvement of the onset potential as well as the kinetics of the electrocatalysis (see Figure 5).

It is worth mentioning that some studies have shown that the presence of transition-metal impurities, particularly iron, can result in inaccurate evaluations of the electrochemical performance.^59−61^ Therefore, to assess the potential effect of Fe-based contamination, activation and LSV measurements of the different Co-based LH samples were repeated in purified and Fe-free KOH on carbon paper electrodes. As illustrated in Figures S4 and S5, the obtained results do not significantly vary from those recorded in unpurified KOH solution. Consequently, the main observed differences in the samples (Figures 4 and 5) can be genuinely attributed to their specific crystallographic structures.

To further rationalize the electrocatalytic performance of these LH structures, DFT + U calculations were employed to address the OER process based on the 4-electron mechanism proposed by Norskov.^62−65^ Theoretically, it is assumed that the water-splitting reaction occurs through a series of successive proton-coupled electron transfer steps, as depicted in the following equations (note: * symbolizes the active surface).
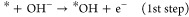

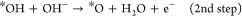

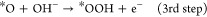

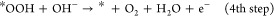
Considering the differences of these LH structures toward the reconstruction into oxyhydroxide phases, the dehydrogenation capability can be a straightforward approximation to address the nature of these active surfaces.^15,66,67^

Herein, it is important to point out some aspects related to the selection and the nature of the active surface. First, recent reports highlight the role of unsaturated cationic centers (*n*-fold-lattice-oxygen-coordinated, with *n* < 6) in nickel hydroxides on the structural and electronic properties that considerably modify the electrocatalytic behavior.^68,69^ Additionally, computational studies on β-NiOOH have demonstrated that the thermodynamics OER overpotential does not depend on the crystallographic facets, and therefore, the OER activity should be uniform across various crystallographic facets on the electrode.^67^ Thus, in order to analyze the inherent chemical reactivity of these Co-based LHs phases, we decided to evaluate the OER mechanism on the defect-free (00*l*) planes, exclusively.

**Figure 6:**
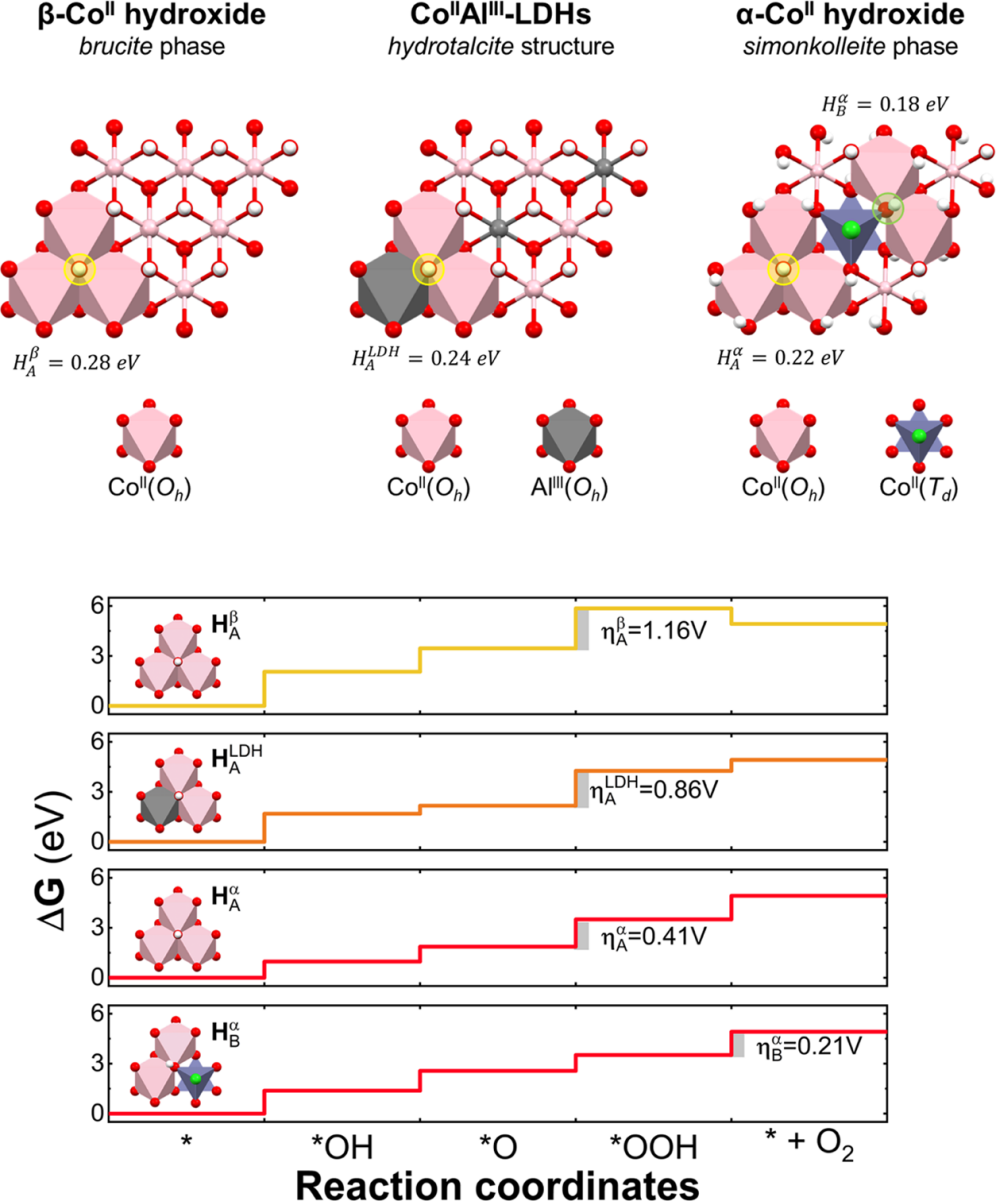
Schematic representation of the (00*l*) plane for LH structures depicting the differences on the OH groups (upper image). *H*_A_ (in yellow) represents an OH group shared by three octahedral cations, while *H*_B_ (in green) denotes an OH group shared by two octahedral and one tetrahedral cations. The *H*_*X*_^*y*^ values represent the calculated DFT + U energies (in eV) for the dehydrogenation process, where *y* and *X* denote the LH structure and the OH type, respectively. Reaction standard free energy diagrams for the OER process, at zero potential (*U* = 0), for each OH type on the LH structures (lower image). The determining steps with the corresponding overpotential values (η) are also depicted in gray color.

A detailed inspection of the (00*l*) surface for the LH family reveals differences in terms of the exposed OH groups, and as a consequence of that, the H atoms involved in the water-splitting reaction. Specifically, in the case of β-LH and LDH structures, only one kind of OH group (denoted as *H*_A_), shared by three octahedral cations, is distinguished. However, in the case of the α-LH phase, besides the aforementioned OH groups, other ones (denoted as *H*_B_) shared by two octahedral cations and one tetrahedral cation are observable (see Figure 6).

First, we decided to explore the free energy calculation of the dehydrogenation reaction (Δ*G*_deh_) for *H*_A_ moieties. The effect of this process over the structures results in the oxidation of Co centers which can be addressed by employing atomic polarization based on Lowdin charges, resembling the oxidation observed in the case of the activation processes (see Figure S6, SI).^66^ The obtained Δ*G*_deh_ values for β-LH, LDH, and α-LH structures are 0.28, 0.24, and 0.22 eV, respectively. These results are in concordance with the observed OER performance. Additionally, in the case of α-LH, the Δ*G*_deh_ values for *H*_B_ moieties (exclusive of simonkolleite-like structures) are 0.18 eV, suggesting that this process is even more favorable through this pathway, as reported by He et al.^15^ and others.^25^ Interestingly, regardless of whether the dehydrogenation process takes place (*H*_A_ or *H*_B_) for the α-LH structure, atomic polarization based on Lowdin charges demonstrates that Co^II^(*O*_h_) sites are the unique Co center that can be oxidized (see Figure S6, SI). Thus, the reconstruction of the α-LH sample into the electrochemically active phase is more favorable for both OH groups. Hence, the observed results from the dehydrogenation point of view are in good agreement with the electrochemical activation process (Figure 4).

Once the active surfaces are defined, we have evaluated the standard Gibbs free energies for each OER’s elementary step in the whole LH family (see DFT + U calculations, OER mechanism). For α-LH, both active surfaces, generated from *H*_A_ and *H*_B_, are considered. In the cases of *H*_A_ active surfaces, the simulations suggest that the superoxide formation (third step) appears as the limiting step, exhibiting Δ*G*_3_ values of 2.39, 2.09, and 1.64 eV for β-LH, LDH, and α-LH structures, respectively (further information can be found in Table S6, SI). For active surfaces generated through the dehydrogenation of *H*_B_ groups, this value decreases to 0.92 eV. Thus, both α-LH active surfaces markedly stabilize the *OOH formation (third step), a crucial step toward OER catalysis (see Figure 6).

Under these conditions, the theoretical thermodynamic overpotential (η_OER_) can be determined by using the following equation
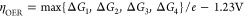
where Δ*G*_*i*_ corresponds to the free energy of each OER step and 1.23 V indicates the equilibrium potential of the OER considering RHE. Thus, theoretical overpotential values of 1.16, 0.86, and 0.41 V for β-LH, LDH, and α-LH (considering *H*_A_ and the third step), respectively, and 0.21 V (*H*_B_ and the fourth step) can be estimated. It is important to remark that the absolute values of computed overpotentials obtained with this methodology should not be directly compared with experimental data because this method has not taken into account the kinetic barriers between intermediates, besides the intrinsic uncertainty associated with DFT + U calculations. However, the OER mechanism’s DFT + U analysis confirms the overall higher activity of the α-LH sample, in good agreement with the experimental electrochemical parameters (see Figure 5). Moreover, we have included the effect of the electrode potential on the OER simulations in Figure S7.

Once demonstrated the superior performance of the α-LH phase from the electrochemical and DFT + U point of view, a thorough ex situ characterization of LH samples has been conducted by in-house Raman spectroscopy, PXRD, and XAS after 5 days at ALBA synchrotron. First, solid samples were deposited on carbon paper (*C*_paper_) electrodes by spray-coating and electrochemically characterized as previously indicated. Analogous trends were detected when comparing these LSVs with the results obtained using glassy carbon electrodes (see Figure S5, SI).

**Figure 7:**
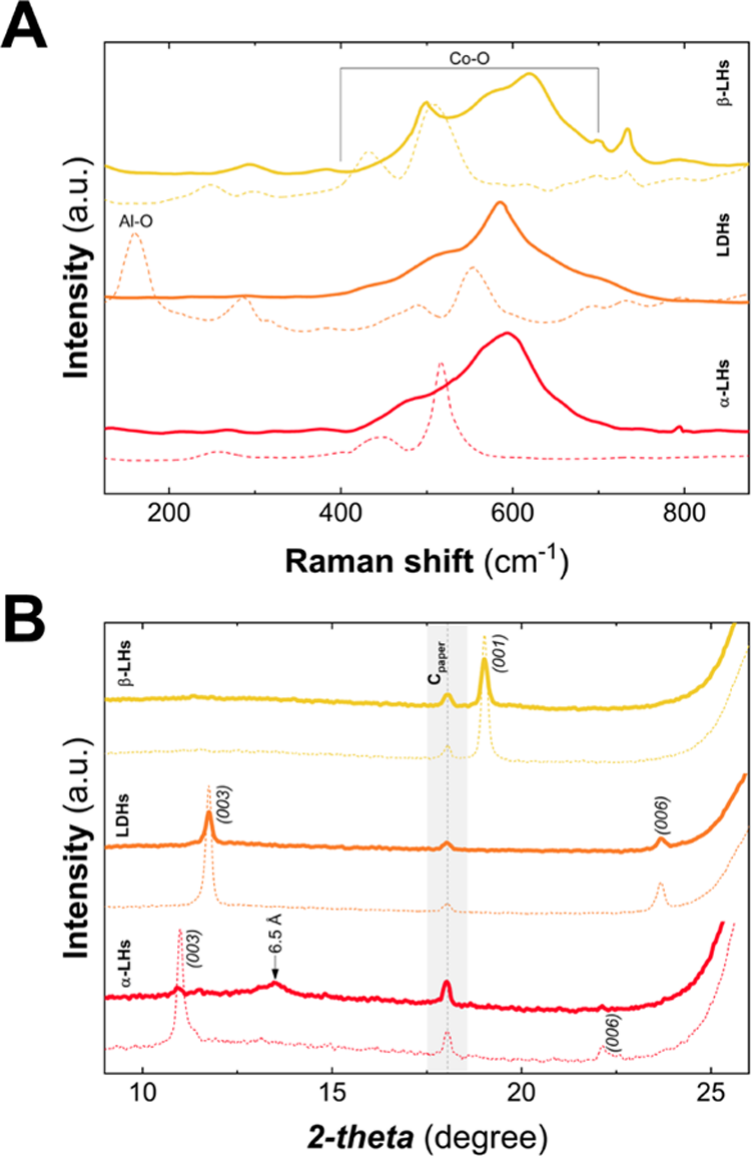
Post-mortem analysis: characterization before (dashed line) and after (solid line) OER catalysis. Raman spectra evidence the surface oxidation of the phases, while in the case of the LDH structure, they confirm the Al dissolution after the OER activity (A). PXRD patterns denote nonchanges in the bulk structure for β-LH and LDH phases, while in the case of the α-LH sample, a massive transformation toward oxyhydroxide-like structure stable under ambient conditions is observable (B). Note: Black dashed line denotes the reflection for the carbon paper substrate (*C*_paper_).

Figure 7A depicts the Raman spectra for the LH family before and after OER measurements on *C*_paper_ electrodes. The main peaks in the range 400–700 cm^–1^ are assigned to M–O vibrations,^70^ previously employed to follow structural changes in LH phases during the OER process.^71^ Herein, in all of the cases, an intense signal centered at ca 600 cm^–1^, attributed to cobalt superoxide species, evidences the partial superficial conversion toward oxyhydroxide-like electroactive phases.^52^ Specifically, in the case of β-LH, the remarkable presence of the signal at 502 cm^–1^ confirms the occurrence of an unreacted material, as expected for nonexpanded hydroxylated phases.^72−74^ On the other hand, the spectrum for the LDH phase exhibits a signal below 200 cm^–1^, typically observed for hydrotalcite-like phases,^75^ which is lost after OER, as a consequence of the partial or complete dissolution of aluminum. It is worth mentioning that this redounds to an increment in the diffusion of the electrolyte into the laminar space.^56^ Finally, in the case of the α-LH sample, the larger Raman shift toward higher energy values could be related to better electrochemical performances.^76^

Considering the partial oxidation of the LH phases as demonstrated by Raman spectroscopy, PXRD patterns measured on the *C*_paper_ electrodes were collected before and after OER catalysis (Figure 7B). Regarding β-LH and LDH samples, nonappreciable differences in the interlayer reflections (00*l*) are observed, apart from subtle changes in the intensity attributable to an inhomogeneous distribution of the solid on the *C*_paper_ electrodes. These results suggest minor structural changes in the nonexpanded β-LH phase that could be understood considering their low activation rate, where the observations carried out by Raman spectroscopy might be related to surface oxidation, exclusively. Note that, in the case of the LDH sample, Raman confirms the complete/total dissolution of Al^III^; thus, if the oxidation of Co is taking place, it would be not be observed by PXRD due to the similar *d*_BS_ values of Co^II^Al^III^ and Co^II^Co^III^ LDH structures.^42,77^ However, in the case of α-LH, the sharp signals attributed to 00*l* reflections vanish completely with the concomitant appearance of a new signal at a 2θ value of around 13.5°.

This new reflection, associated with a distance ca 6.5 Å, represents the first indication of a stable electroactive material associated with the formation of a layered oxyhydroxide structure. This is particularly relevant if we consider that for LDHs—as recently reported by several in-operando studies—the active phase is generated concomitantly with the applied potential, disappearing under open potential circuit conditions (*V* = 0). Therefore, this result suggests a massive and permanent transformation of the α-LH toward a new highly electroactive (oxy)hydroxide-like structure,^73^ in agreement with the higher activation process (Figure 4) and the considerably better electrochemical performance (Figure 5).

**Figure 8:**
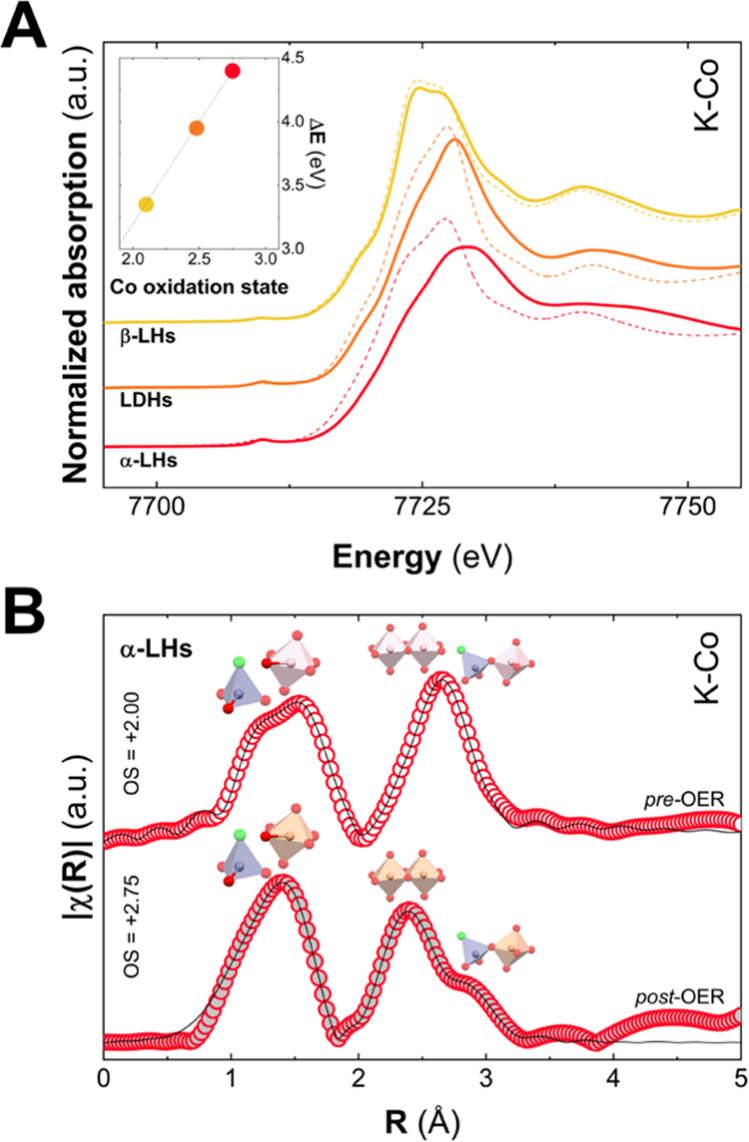
Normalized XANES spectra at the Co K-edge, before (dashed lines, pre-OER) and after (solid lines, post-OER) the OER measurements (A). Final oxidation state calculated by Capehart’s method after OER measurements and the calibration curve (A—inset). Pre- and post-OER Fourier transform of the extracted κ^2^-weighted EXAFS oscillations for the measured samples—circles—and their corresponding fittings—black line—for the α-LH sample (B). First peaks are attributed at Co–O distances: blue for Co^II^(*T*_d_), pink for Co^II^(*O*_h_), and orange for Co^III^(*O*_h_). Second peaks consider the Co–Co distances, polyhedrons represent pink–pink for Co^II^(*O*_h_)–Co^II^(*O*_h_), blue–pink for Co^II^(*T*_d_)–Co^II^(*O*_h_), orange–orange for Co^III^(*O*_h_)–Co^III^(*O*_h_), and blue–orange for Co^II^(*T*_d_)–Co^III^(*O*_h_). In all of the cases, the XAS spectra are represented without phase correction.

To shed light on the changes taking place in the LH samples during the OER catalysis, ex situ XANES spectra were recorded (Figure 8A). An almost unnoticeable shift in the position of the absorption edge for β-LH is observed, while a slight one appears for the LDH phase. Nevertheless, in the case of α-LH, the shift becomes evident. The use of the inflexion point through the derivative gives a good first estimate of the valence state of a sample; nevertheless, a more suitable method for the quantification of the final oxidation state is provided by the integral method as described by Capehart et al.^78^ Indeed, the calculated final oxidation states are 2.10, 2.48, and 2.75 for β-LHs, LDHs, and α-LHs, respectively, pointing out the superior reactivity of α-LHs toward the oxidation and stabilization of higher oxidation states on Co centers (Figure 6A—inset). It is worth mentioning that this high oxidation state in Co-based LHs has been exclusively reported under in situ experiments at anodic potentials higher than 1.6 V vs RHE.^74^

Moreover, to unveil the nature of this oxidized α-LH phase, the EXAFS region was analyzed (Figure 8B). Figure S8 compiles the data for the LDH sample. At first glance, in the case of the α-LH sample after OER catalysis, the shortening in the peaks attributed to the first and the second shell confirms significant structural changes triggered during the electrocatalytic activity. To quantify this information, a model employing a Co–O and two Co–Co coordination shells is proposed (Table S7), for nonoxidized and oxidized Co environments. The fitting confirms the lattice compression due to the presence of Co^III^ cations, as expected according to the cationic sizes: Co_*T*_d__^II^ = 0.580 Å, Co_*O*_h__^II^ = 0.745 Å, and Co_*O*_h__^III^ = 0.610 Å. Specifically, in the case of the first shell, the observed distance at 1.9 Å is now compatible with the presence of Co^II^(*T*_d_)–O and Co^III^(*O*_h_)–O, while the lack of the distance at 2.1 Å suggests the absence of Co^II^(*O*_h_). On the other hand, it is possible to observe two Co–Co distances at 2.8 and 3.1 Å, ascribable to Co^III^(*O*_h_)–Co^III^(*O*_h_) and Co^II^(*T*_d_)–Co^III^(*O*_h_), respectively. Thus, this would be an indication that Co^II^(*T*_d_) environments are still present, while those Co-located in *O*_h_ sites are more prone to be oxidized during the water-splitting process, as anticipated by DFT + U calculations (Figure S6), resulting in the formation of a new Co(III)-based oxyhydroxide-like phase.^52,79^

**Figure 9:**
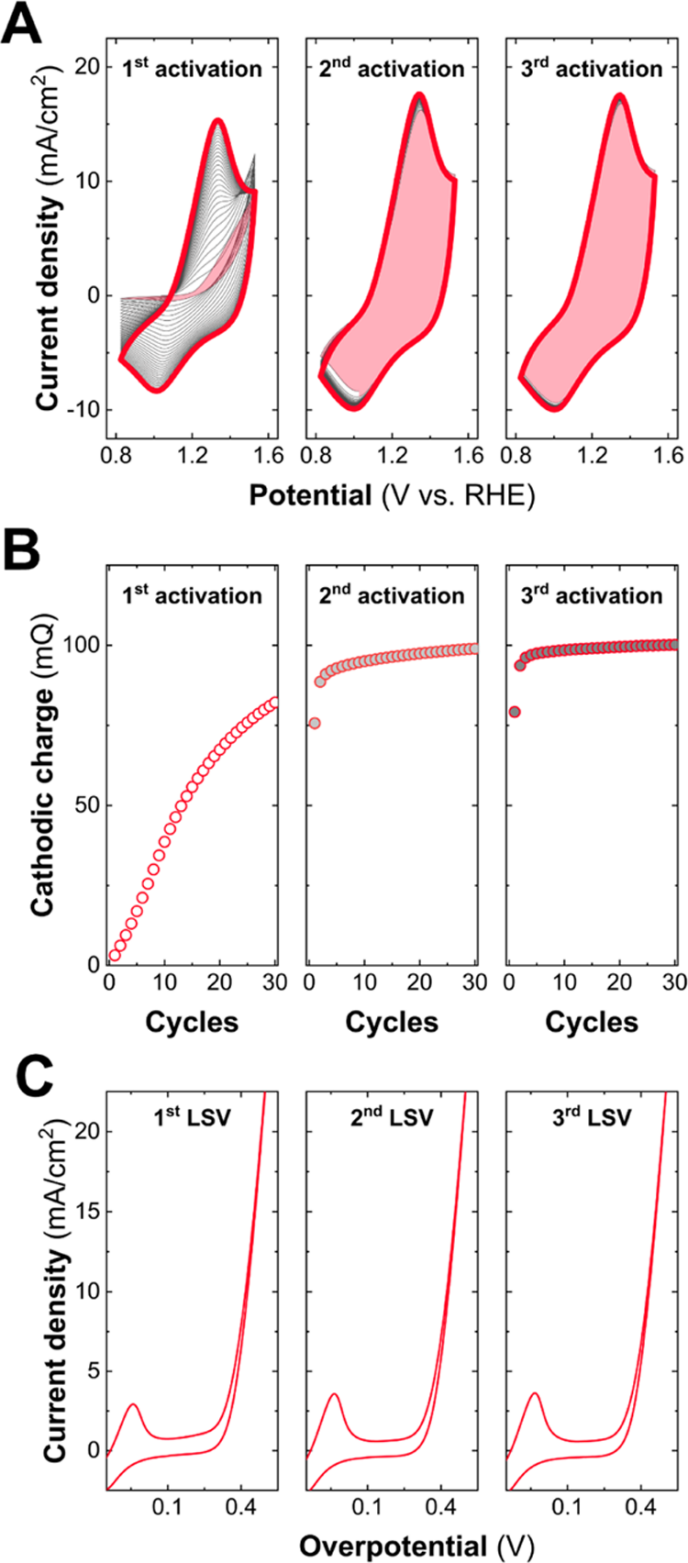
Intermittent electrochemical activation experiments. First row: activation processes consisting of 30 cyclic voltammetry curves performed in a 1 M KOH aqueous solution at 50 mV/s for the α-LH structure. The first cycles are depicted as shading curves, and the final ones are presented by thick colored lines (A). Between each activation cycle, LSVs at 5 mV/seg and a 10 min lapse time were applied. Evolution of the cathodic charge during the activation time cycles (B). LSVs performed after each activation process (C).

Interestingly, this oxyhydroxide-like structure is highly stable under ambient conditions, as demonstrated by characterizing samples more than one month after OER catalysis. In fact, this relevant stability and, therefore, the tendency toward Co^III^ stabilization are key aspects when performing electrochemical measurements, becoming evident in a series of intermittent activation cycles and consecutive LSV curves, as shown in Figure 9. Analogous measurements for the other LH members are presented in Figure S9. In this regard, the superior electrochemical stability of the α-LH, especially with respect to the nonexpanded LH, can also be noticed when performing 48 h ON–OFF chronopotentiometry at 10 mA/cm^2^ (Figure S10). It is worth noting that the resistances observed in the EIS measurements (Figure S11) after the long-term stability OER test decreased significantly in all cases, likely due to the formation of oxyhydroxide species. However, despite this reduction in the resistance values (all of the semicircles are reduced), the required overpotential to maintain a current density of 10 mA/cm^2^ is increased, suggesting that this effect is mainly related to a progressive loss of the electrocatalyst from the electrode surface during the water oxidation experiments rather than the inactivation of their electroactive centers. Additionally, Raman spectroscopy suggests the presence of Co_3_O_4_ on all samples, as can be observed by the appearance of well-defined and characteristic signals between 180 and 670 cm^–1^ (DOI 10.1088/0022-3719/21/7/007). However, it should be noted that this presence is only superficial as it was not detected by PXRD analysis. As previously observed in the post-mortem characterization, only the α-LH phase is massively transformed into an oxyhydroxide-like phase. Indeed, the PXRD patterns after the long-term stability tests (Figure S13) show an even more intense peak corresponding to a distance of 6.8 Å, which is exclusively observed for the α-LH sample, indicating the continuous formation of the stable electroactive oxyhydroxide structure. Consequently, the α-LH phase surpasses the other Co-based LH structures during the whole set of experiments, highlighting both superior OER performance and electrochemical stability along the water oxidation reaction.

## Conclusions

In this work, we have conducted a comprehensive study in order to shed light on the influence of the crystallographic structure of the most important Co-based layered hydroxides on the water oxidation performance. Thus, we have systematically characterized the Co-based β-LH, LDH, and α-LH phases, providing clear fingerprints for their straightforward identification as well as an electronic description based on DFT + U calculations. The α-LH phase, containing both octahedral and tetrahedral cobalt sites, exhibits a superior OER catalytic behavior with significantly lower overpotentials and Tafel slopes, accompanied by higher TOFs values, compared to the other structures, highlighting a key role of the chemical nature for the LH structure. Additionally, by the combination of structural, spectroscopical, and theoretical studies, we demonstrate that α-LH favorably reconstructs into an electrochemically active Co(III)-based oxyhydroxide-like phase, with a market higher reactive behavior of both OH groups for the α-LH. Indeed, this is also manifested by the identification of permanent and stable under ambient conditions of a highly active oxyhydroxide-like phase after the OER process for the first time.

Overall, this work illustrates the importance of precisely controlling the crystalline structure and the electronic properties of layered hydroxides, which is a key parameter governing the OER process. Certainly, this allows the design of more efficient electrocatalysts based on a new family of layered hydroxides, capable of surpassing the benchmark materials reported to date, such as the LDHs. We envision promising results by precisely tuning the electronic properties by modification in the covalent coordinated anion and also by judicious incorporation of Fe cations into α-LH structures. These works are in progress.

## Supporting Information Available

Synthetic conditions; TEM inspection; structural parameters obtained from PXRD analysis; FTIR and UV–vis spectroscopic signals; structural parameters obtained from XAS fittings; cathodic charge evolution; polarization based on Lowdin charges; DFT + U-calculated Δ*G* values for the 4-step OER mechanism and reaction standard free energy diagrams for the OER process at different potentials; electrochemical characterization on carbon paper; pre- and post-OER EXAFS analysis for the LDH sample; structural parameters obtained from XAS fittings for the sample α-LH after OER catalysis; intermittent electrochemical activation experiments for samples β-LH and LDH; long-term stability test; EIS measurements before and after the stability test; Raman spectroscopy and PXRD characterization before and after water oxidation; and long-term ON–OFF stability test (PDF)
