# Supplementary material for: Crystallographic and Geometrical Dependence of Water Oxidation Activity in Co-Based Layered Hydroxides
Source: ACS Catal. 2023 Jul 24;13(15):10351–63. doi: 10.1021/acscatal.3c01432 (PMC10407849; doi:10.1021/acscatal.3c01432)
Supplement: Supplementary file 1 — cs3c01432_si_001.pdf [file cs3c01432_si_001.pdf]

Supplementary Information

Crystallographic and geometrical dependence of water oxidation activity in Co-based layered hydroxides

Roger Sanchis-Gual,[1,a] Diego Hunt,[2,a] Camilo Jaramillo-Hernández,[1] Alvaro Seijas-Da Silva,[1] Martín Mizrahi,[3,4] Carlo Marini,[5] Víctor Oestreicher,[1,*] Gonzalo Abellán[1,*]

[1] Instituto de Ciencia Molecular (ICMol), Universidad de Valencia, Catedrático José Beltrán 2, 46980, Paterna, Valencia, Spain.

[2] Departamento de Física de la Materia Condensada, GIyA. Instituto de Nanociencia y Nanotecnología, CNEA-CAC-CONICET. Av. Gral. Paz, 1650, San Martín, Buenos Aires, Argentina.

[3] Instituto de Investigaciones Fisicoquímicas Teóricas y Aplicadas (INIFTA), Departamento de Química, Facultad de Ciencias Exactas. Universidad Nacional de La Plata, CCT La Plata- CONICET. Diagonal 113 y 64, 1900, La Plata, Argentina.

[4] Facultad de Ingeniería, Universidad Nacional de La Plata. Calle 1 esq. 47, 1900, La Plata, Argentina.

[5] CELLS−ALBA Synchrotron, Cerdanyola del Vallès, 08290, Barcelona, Spain.

[a] Both authors contributed equally to this work.

Corresponding authors' email: victor.oestreicher@uv.es, gonzalo.abellan@uv.es

Experimental section

Chemicals. Aluminium chloride hexahydrate ($AlCl_3.6H_2O$), cobalt chloride hexahydrate ($CoCl_2.6H_2O$), urea, hexamethylenetetramine (HMT), glycidol (Gly), acetylene black, and Nafion (117 solution) were purchased from Sigma-Aldrich. Potassium hydroxide KOH, 99.98 % (CAS number: 1310-58-3, Lot: A0440930) and ethanol absolute (EtOH) were purchased from Panreac. All chemicals were used as received. Milli-Q water was obtained from Millipore Milli-Q equipment.

Co-based LH synthesis

Samples were obtained through specific synthetic protocols for each phase keeping in mind the obtaining of micrometrical hexagonal crystals (see Figure S1, in SI). In all the cases, solids were separated for the mother liquors by filtration, washed three times with water, water:ethanol mixture and finally with EtOH. Samples were dried at room temperature and kept in desiccators for further characterization.

Synthesis of β-LH. β-Co sample was synthesised by employing HMT as an alkalinisation reagent following the protocol reported by Liang *et al.*[1] Typically, β-LH precipitation is driven by the hydrolysis of HMT at *ca.* 97 ºC for 5h, under an inert atmosphere, in an aqueous solution containing initial concentrations fixed to: [MCl_2] = 7.5 mM and [MHT] = 45 mM.

Synthesis of LDH. CoAl LDH was synthesised by employing urea as an alkalinisation reagent following the protocol reported by Liu *et al.*[2] Typically, LDH precipitation is driven by the hydrolysis of urea at *ca.* 97 ºC for 48 h, under inert an atmosphere, in an aqueous solution containing initial concentrations fixed to: [MCl_2] = 10 mM, [$AlCl_3$] = 5 mM, [urea] = 35 mM. In the case of NiAl LDHs the urea concentration was increased to 70 mM.

Synthesis of α-LH. α-Co LH was synthesised by employing the *Epoxide Route*[3,4] following the protocol reported by Arencibia *et al.*[5] Typically, precipitation is driven by the reaction taking place between chloride and Gly at room temperature for 48h, in an aqueous solution containing initial concentrations fixed to: [MCl_2] = 10 mM, [NaCl] = 80 mM, [Gly] = 400 mM.

Chemical and Structural Characterization

Powder X-ray powder diffraction (PXRD) patterns were obtained employing a PANalytical Empyrean X-ray platform with a capillary platform and copper radiation (Cu Kα = 1.54178 Å). Measurements were carried out by triplicate in the 2-theta range 2−70° by employing a step size of 0.02 °/step with an integration time of 1 s.

Attenuated total reflectance-Fourier transform infrared spectroscopy (ATR-FTIR) spectra were collected in a Bruker alpha II FTIR spectrometer in the 4000−400 cm^{-1} range.

UV-Vis absorption spectra of the solid samples were recorded in a reflectance mode employing a Jasco V-670 spectrometer.

Individual point Raman spectra were carried out using a Horiba LabRAM HR evolution, employing a blue laser (473 nm) in the 100–1000 cm^{-1} range. A 50x Objective with a 600 mm^{-1} grating was employed to acquire all Raman spectra. Measurements were performed at least five times at 1.25 mM of laser power, with an acquisition time of 20 s.

X-ray absorption spectroscopy

X-ray Absorption Spectroscopy (XAS) measurements were performed at the BL-22 (CLÆSS) beamline from the ALBA synchrotron (Barcelona–Spain). XANES+EXAFS Co K-edge spectra were measured at room temperature in transmission mode. Absorbents of as-synthesised fresh samples were prepared from fresh powder samples in pellets of 1.3 mm diameter and

sealed with Kapton® tape (50 µm in thickness) to prevent the oxidation of the sample. The optimum amount of material for the measurements was calculated by the program hephaestus which is part of the Demeter package.[6] The pre and post-OER samples were measured over carbon paper electrodes before and after the OER catalysis. A Si(111) double-crystal monochromator was used to obtain a monochromatic incident beam over the sample, and the intensities of the incident and transmitted X-rays were measured using two ionization chambers, respectively. XAS spectra were collected from 7590-8550 eV with a reduced step (0.3 eV) in the XANES region (7690 to 7750 eV) for Co K-edge. The incident photon energy was calibrated using the first inflection point of the Co K-edge (7709 eV) from reference foils of metallic Co. For each sample, three spectra were taken with exposure times of 4 min for each one to later be averaged. XANES data treatment was performed by subtracting the pre-edge background followed by normalisation by extrapolation of a quadratic polynomial fitted at the post-edge region of the spectrum using ATHENA AUTOBK background removal algorithm.[7] The quantitative analysis of the EXAFS results were performed by modelling and fitting the isolated EXAFS oscillations. The EXAFS oscillations χ(k) were extracted from the experimental data with standard procedures using the Athena program. The k^2 weighted χ(k) data, to enhance the oscillations at higher k, were Fourier transformed. The Fourier transformation was calculated using the Hanning filtering function. EXAFS modelling was carried out using the ARTEMIS software.[6] Theoretical scattering path amplitudes and phase shifts for all paths used in the fits were calculated using the FEFF9 code.[8] The k-range was set from 2.5 to 12.3 $Å^{-1}$. The passive reduction factor S_0^2 values were restrained to 0.8 for Co, respectively. These values were obtained from the fitting standard foils of metallic Co and constraining the coordination numbers to those corresponding to each structure.

Electrochemical Characterization

KOH purification. To purify the electrolyte (KOH), Ni fibers (BEK-POR 2N118-0.25, Bekaert. 99.9% purity) were used as both working and counter electrodes for a prolonged electrolysis process lasting 1 day at high current densities. This approach was motivated by previous reports.[9,10]

Electrode Preparation. For the electrode preparation, a dispersion composed of 2,5 mg of powder material, 1 mL of water and ethanol (1:1) and 7 µL of Nafion (5 %) was sonicated in order to obtain a well-dispersed suspension. Then, 5.7 µL was drop-casted in a previously polished (sequentially with 1.0, 0.3 and 0.05 µm alumina powder) 3 mm glassy carbon electrode. Afterwards, the solvent was let evaporated at room temperature. The electrode mass loading achieved was around 0.20 $mg \cdot cm^{-2}$. On the other hand, modified carbon paper electrodes were prepared by spray coating the previous dispersion (using an airbrush from Harder Evolution) on carbon papers with a geometrical area of 2 x 1 cm^2. The electrode mass loading achieved was around 0.50 $mg \cdot cm^{-2}$.

Electrochemical Measurements.

Electrochemical tests were performed in a three-electrode cell equipped with glassy carbon acting as the working electrode and a platinum wire as the counter electrode. As the reference electrode, a silver-silver chloride (Ag/AgCl (3 M KCl)) was used. All potentials were converted referring to the oxygen evolution reaction overpotential. The measurements were performed on an Autolab PGSTAT 128N potentiostat/galvanostat. Linear sweep voltammetry (LSV) measurements were carried out at 5 $mV \cdot s^{-1}$ in a previously N_2 purged 1 M KOH aqueous solution. Prior to this, 30 cyclic voltammetry measurements were performed at 50 $mV \cdot s^{-1}$ to activate the material.

Similar conditions were used to carry out the measurements on modified carbon paper electrodes. However, in this case, another carbon paper with higher surface area (3 x 3 cm^2) was used as the counter electrode.

Electrochemical surface area was acquired by measuring the current associated with double-layer capacitance from the scan rate dependence of CVs. The potential range used for the CVs was from -0.25 to -0.05 V versus Ag/AgCl (3 M KCl). The scan rates were 300, 250, 200, 150, 100 and 50 mV·s^{-1}. The double layer capacitance was estimated by plotting the (j_a–j_c) (anodic versus cathodic currents) at –0.15 V versus Ag/AgCl (3 M KCl) against the scan rate. The ECSAs were measured on the working electrodes after performing an activation process consisting of 10 CVs at 50 mV·s^{-1} around their redox processes.

The turnover frequency (TOF) values were calculated from the following equation:

$$TOF = jA\ /\ 4Fn$$

where j is the current density at a given overpotential, A is the surface area of the working electrode, F is the Faraday constant, and n is the total number of moles of the material.

Electrochemical impedance spectroscopy (EIS) measurements were carried out using a Gamry 1000E potentiostat/galvanostat controlled by Gamry software by applying an AC amplitude of 10 mV in the frequency range of 100–105 Hz at an overpotential of 0.40 V. EIS data were analysed and fitted by means of Gamry Echem Analyst v. 7.07 software.

Electrochemical stability tests were done using an Autolab PGSTAT 128N potentiostat/galvanostat. At first, samples were subjected three times to 30 activation cycles, an LSV and an OFF time of 10 min.

DFT+U calculations

All calculations were performed in periodic boundary conditions employing density functional theory (DFT) as implemented in the Quantum Espresso code,[11] which is based on the pseudopotential approximation to represent the ion-electron interactions, and plane waves basis sets to expand the Kohn-Sham orbitals. Ultrasoft-type pseudopotentials were adopted, in combination with the PBE formalism to compute the exchange-correlation term.[12] The magnetic states are described through the Kohn-Sham Hamiltonian in the framework of spin-polarized calculations, plus a Hubbard term. On the basis of our previous reports the incidence of the Hubbard parameter in the DFT+U calculations on the magnetic coupling and other properties was fixed to 4.5 eV for Co atoms.

In all cases, spin-orbit contributions were considered.[13] An energy threshold of 10-8 au was used for self-consistency, while for geometry optimization the convergence criteria were 10-6 au for the energy and of 10-3 au for the forces per atom. To improve the numerical convergence a first-order Methfessel-Paxton spreading was implemented. van der Waals interactions were considered by including the semiempirical correction DFT-D originally introduced by Grimme[14] and implemented in a plane-wave framework by Barone and co-workers.[15] The simulations were carried out on supercells with specific ordering of the metal polyhedra within the layers. Brillouin zone sampling was performed on these supercells with a Monkhorst-Pack grid, checking for convergence with respect to the number of k-points. A 6x6x1 k-point grid was used in both cases. The atomic structures reported in this work were visualised using XCrysDen.[16]

OER mechanism. We employed the computational hydrogen electrode (CHE) method developed by Nørskov.[17] It assumes the chemical potential of a proton-electron pair equals that of gas-phase H2 in standard condition, G(H++e) =G(H2). To each step of the OER mechanism we estimated the Gibbs free energy change, ΔG=ΔE+ZPE−TΔS at a standard

condition where ΔE is the DFT+U total energy of the system substrate + adsorbate, ZPE is the zero-point energy18 and the last term correspond to the entropic correction calculated at a temperature of 298.15 K. In this framework, the global reaction $4OH^- \rightarrow 2H_2O + O_2 + 4e^-$ involves a standard Gibbs free energy change of 4.92 eV at room temperature. Harmonic approximation was employed to treat the allowed vibrations and the molecular partition equation of statistical mechanics under standard conditions was used to obtain the entropic corrections. The potential bias effect was included on all states involving an electron in the electrode, by $\Delta G_U = -eU$, where U is the electrode potential.

The reaction free energy is then calculated as

$$\Delta G(U, p_{H2}=1bar, T) = \Delta G + \Delta G_U$$

The reference energy for the gaseous O_2 was corrected to yield the calculated total energy sum of the OER pathway as 1.229 eV per electron (4.92 eV to the global reaction),[19] because of the well-known difficulties in obtaining accurate energy of open-shell triplet O_2 within DFT.[20]

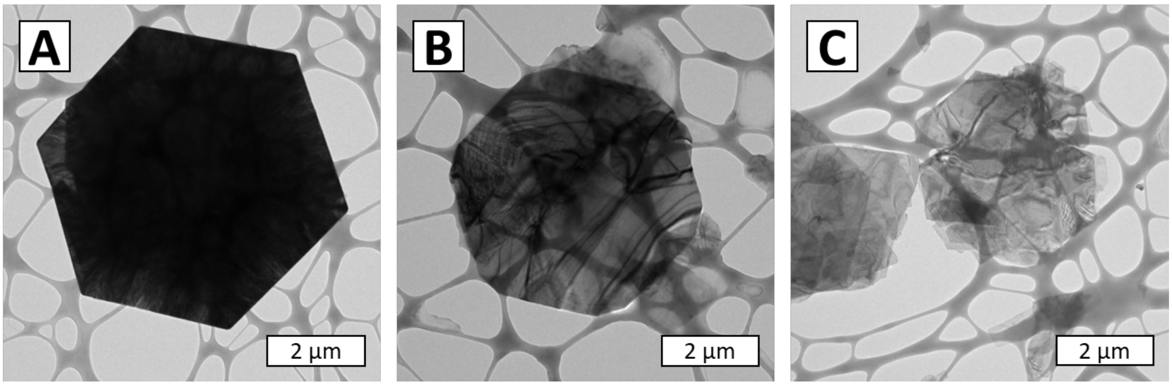

Figure S1. TEM inspection of the Co-based LH samples, β-LH (A), LDH (B) and α-LH (C).

Table S1. Crystallographic parameters of the Co-based LHs family.

Sample	d_{BS} (Å)	a (Å)
β-LH	4.66	3.18
LDH	7.52	3.06
α-LH	8.04	3.14

Table S2. FTIR signals of the Co-based LHs family.

Sample	FTIR signal (cm^{-1})					
	ν(O-H)	δ(H_2O)	ν(CO_2)	δ(CO_2)	δ(M-O-H)	ν(M-O)
β-LH	3620	-	-	-	432	-
LDH	3354	1572	1345	778	550	412
α-LH	3465	1605	-	-	605	485

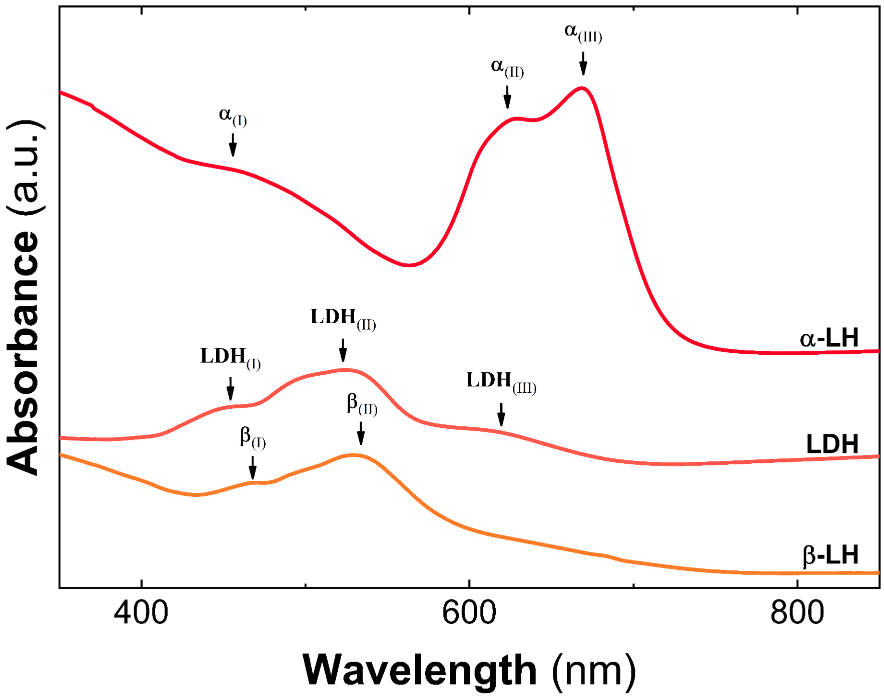

Figure S2. UV-Vis spectra of the Co-based LHs family highlighting the position of the peaks (see Table S3 for further details).

Table S3. UV-Vis signals of the Co-based LHs family as presented in Figure S2.

Sample	Signal	Environment (nm)	
		O_h	***T_d***
β-LH	$\beta_{(I)}$	468	-
	$\beta_{(I)}$	529	-
LDH	$LDH_{(I)}$	453	-
	$LDH_{(II)}$	524	-
	$LDH_{(III)}$	668	-
α-LH	$\alpha_{(I)}$	452	
	$\alpha_{(II)}$	-	629
	$\alpha_{(III)}$	-	668

Table S4. Structural results from the EXAFS fitting (N: coordination number, R: interatomic distance and σ^2: Debye-Waller factor) at the Co K-edge for the Co-based LHs family.

**Sample**	***1st shell***						***2nd shell***					
	1st environment			***2nd environment***			***1st environment***			***2nd environment***		
	N	***R*** (Å)	σ^2(Å^2)	***N***	***R*** (Å)	σ^2(Å^2)	***N***	***R*** (Å)	σ^2(Å^2)	***N***	***R*** (Å)	σ^2(Å^2)
β-LH				5.9	2.09	0.008	6.1	3.15	0.06			
LDH				6.0	2.10	0.007	4.0	3.12	0.008	1.9	3.09	0.007
α-LH	0.9	1.91	0.009	4.3	2.11	0.007	5.9	3.11	0.009			

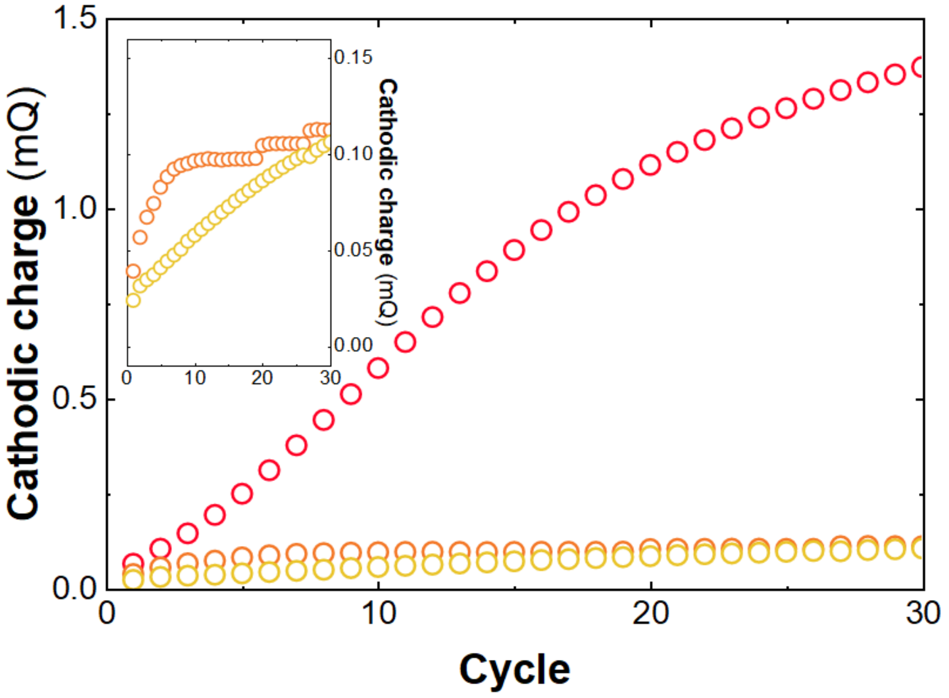

Figure S3. Evolution of the cathodic charge of the different Co-based LH structure during the activation processes carried out before the OER experiments. The activation was performed in a 1 M KOH aqueous solution at 50 mV/s.

Table S5. Electrochemical values of the different Co-based LH structures. Results extracted from Figures 4 and 5.

Sample	OP@10 mA/cm^2 (mV)	Tafel slope (mV·dec^{-1})	ECSA (mF/cm^2)	R_{OER} (Ω)	Cathodic charge, 30th cycle (mQ)
β-LH	547	110	41.5	600	0.106
LDH	508	101	44.6	480	0.112
α-LH	460	78	56.5	130	1.374

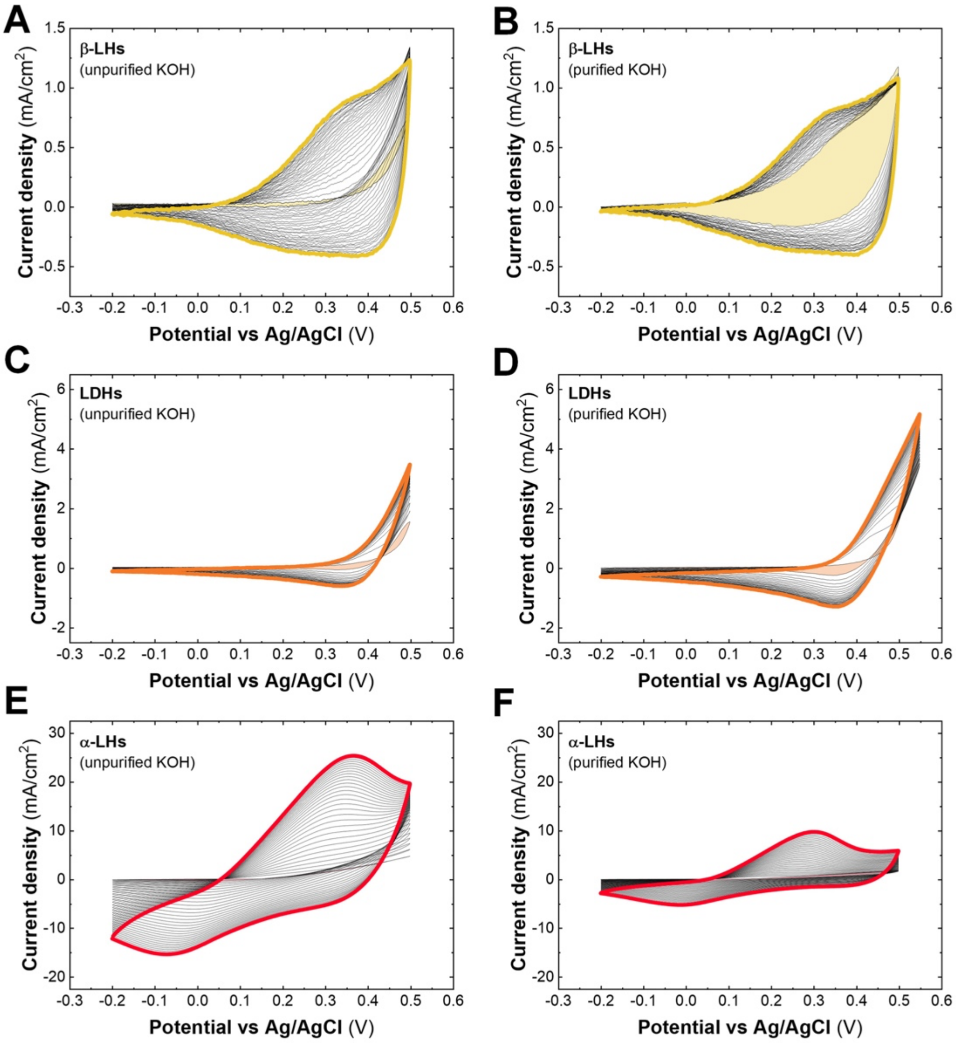

Figure S4. Activation processes carried out before the OER experiments consisted of 30 cyclic voltammetry curves performed in a unpurified (left) and purified (right) 1 M KOH aqueous solution at 50 mV/s for each Co-based LH structure: β-LH (A, B), LDH (C, D) and α-LH (E, F). The first cycles are depicted as shading curves, the final ones are presented by thick coloured lines.

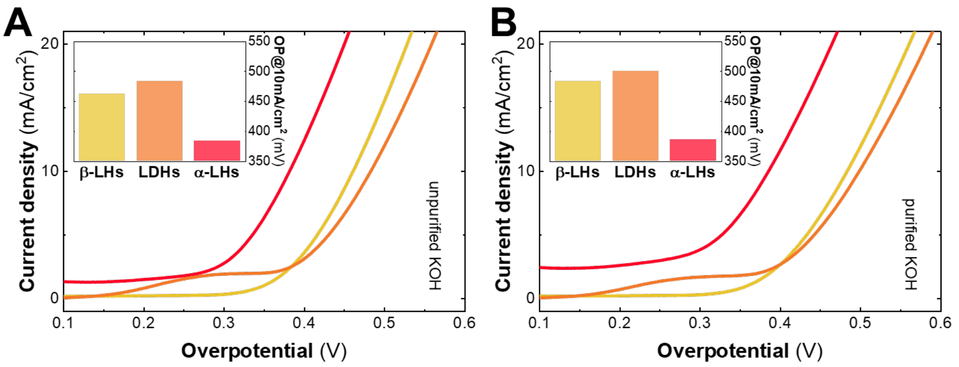

Figure S5. Electrochemical characterization for each Co-based LHs structure was recorded on a carbon paper electrode collector. Linear sweep voltammetry curves were measured at 5 mV/s in unpurified (left-A) and purified (right-B) 1 M KOH aqueous solution. Inset: Overpotential values required for a current density of 10 mA/cm^2.

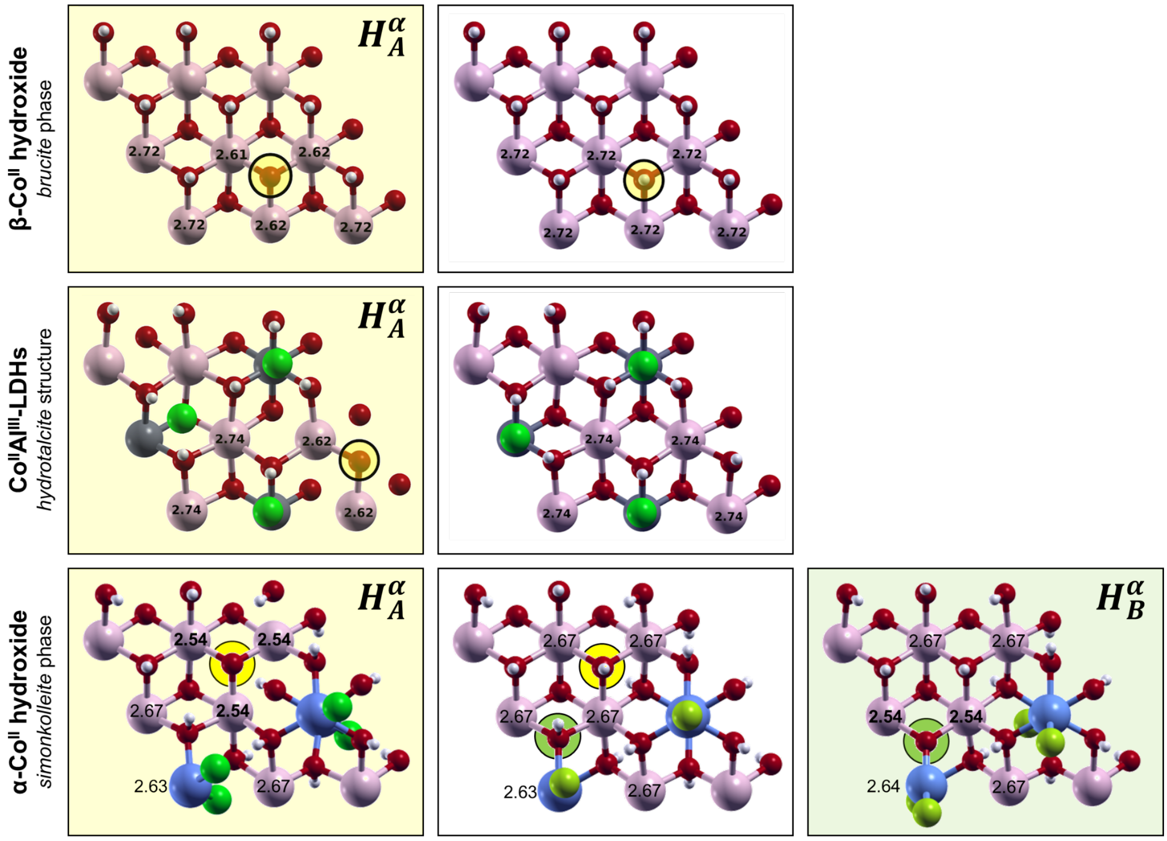

Figure S6. Polarization (P) based on Lowdin charges for native and active surface structures for the Co-based LHs phases considering both H_A and H_B sites. In the case of α-LH structure, the decrease of P on the octahedral cobalt ions suggests that the activation of the surface promotes the oxidation of the $Co^{II}(O_h)$ atoms instead of the $Co^{II}(T_d)$ ones for both H sites.

Table S6. DFT+U calculated ΔG values (in eV) for the 4-step OER mechanism.

**Structure**	**β-LH**	**LDH**	**α-LH**	
**Step**	**H_A**	**H_A**	**H_A**	**H_B**
1st step	2.04	1.69	0.97	1.38
2nd step	1.41	0.48	0.89	1.20
3rd step	2.39	2.09	1.64	0.92
4th step	-0.93	0.66	1.42	1.43

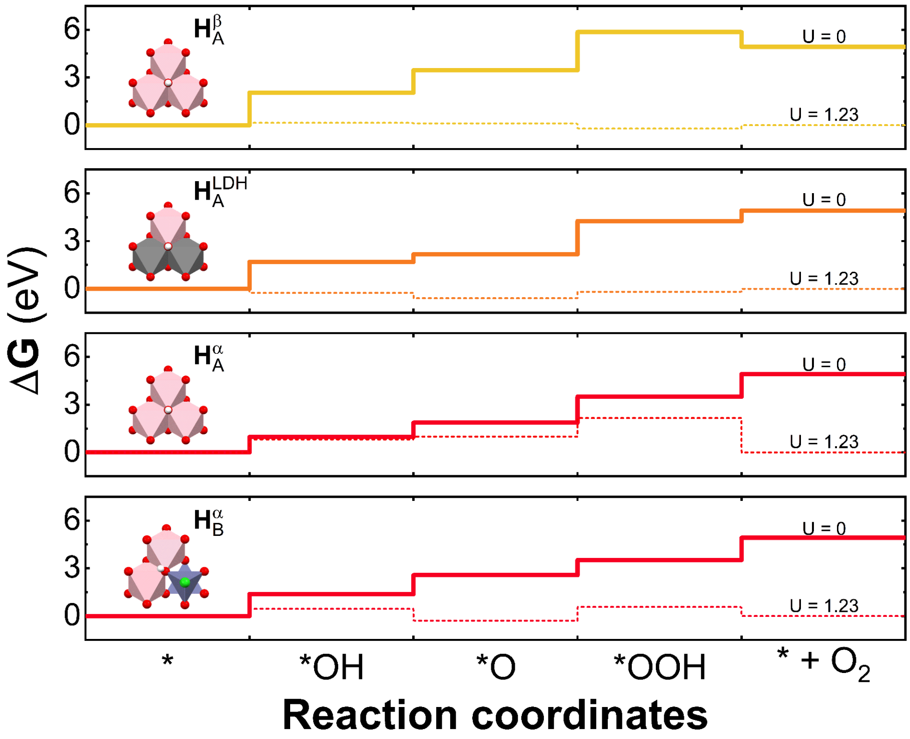

Figure S7. Reaction standard free energy diagrams for the OER process, at zero potential (U = 0, filled lines) and equilibrium potential for oxygen evolution (U = 1.23, dashed lines), for each OH type on the LHs structures. These results show that equilibrium potential (1.23) is not enough for all steps to be thermodynamically favourable.

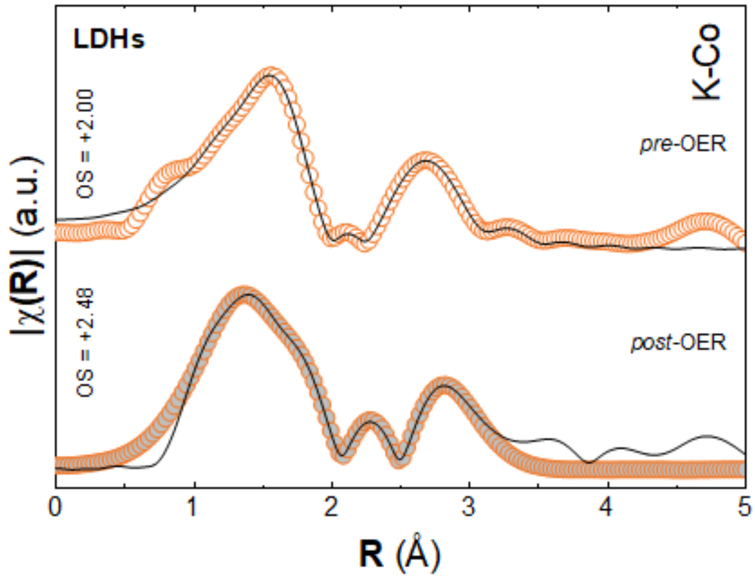

Figure S8. Pre- and post-OER Fourier transform of the extracted κ^2-weighted EXAFS oscillations for the LDH sample –circles– and their corresponding fittings –black line–.

Table S7. Structural results from the EXAFS fitting (N: coordination number, R: interatomic distance and σ^2: Debye-Waller factor) at the Co K-edge for the Co-based α-LH after OER catalysis.

**Sample**	***1st shell Co-O***						***2nd shell Co-Co***					
	1st environment			***2nd environment***			***1st environment***			***2nd environment***		
	N	***R*** (Å)	σ^2(Å^2)	***N***	***R*** (Å)	σ^2(Å^2)	***N***	***R*** (Å)	σ^2(Å^2)	***N***	***R*** (Å)	σ^2(Å^2)
α-LH (*post* OER)	5.1	1.9	0.007				3.8	2.82	0.006	1.4	3.1	0.008

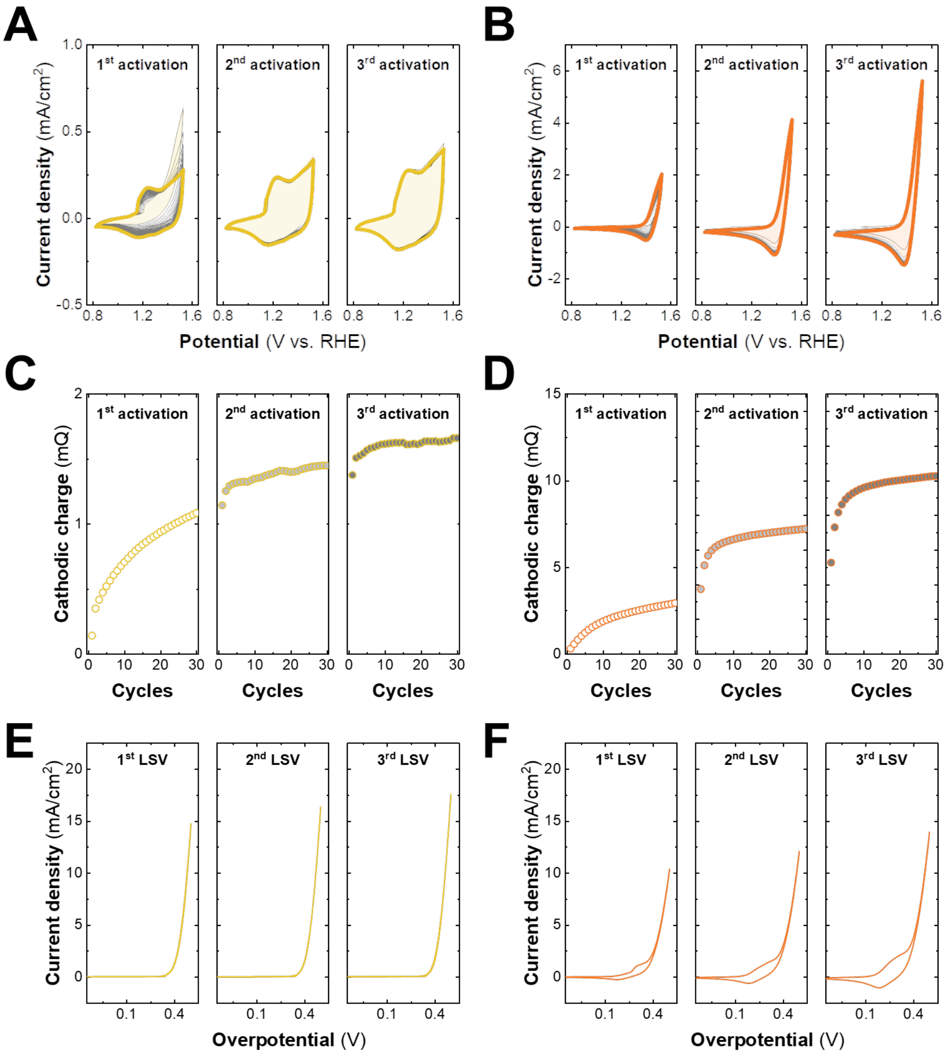

Figure S9. Intermittent electrochemical activation experiments. First row: activation processes consisting of 30 cyclic voltammetry curves performed in a 1 M KOH aqueous solution at 50 mV/s for β-LH (left) and LDH (right) samples. The first cycles are depicted as shading curves, the final ones are presented by thick coloured lines (A and B). Between each activation cycle, LSVs at 5 mV/seg and a 10 min lapse time were applied. Second row: evolution of the cathodic charge during the activation time cycles (C and D). Third row: LSVs performed after each activation process (E and F).

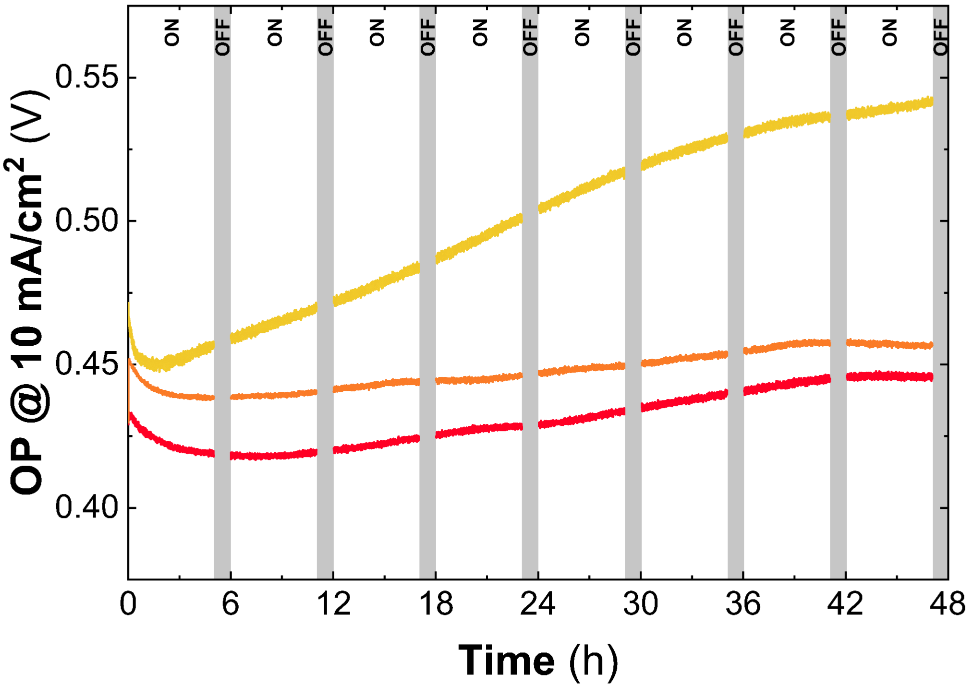

Figure S10. Long-term ON-OFF stability tests performed by chronopotentiometry at 10 mA/cm^2 of the different LH samples recorded on a carbon paper electrode collector in 1 M KOH aqueous solution (5h ON + 1h OFF). β-LH (yellow), LDH (orange) and α-LH (red).

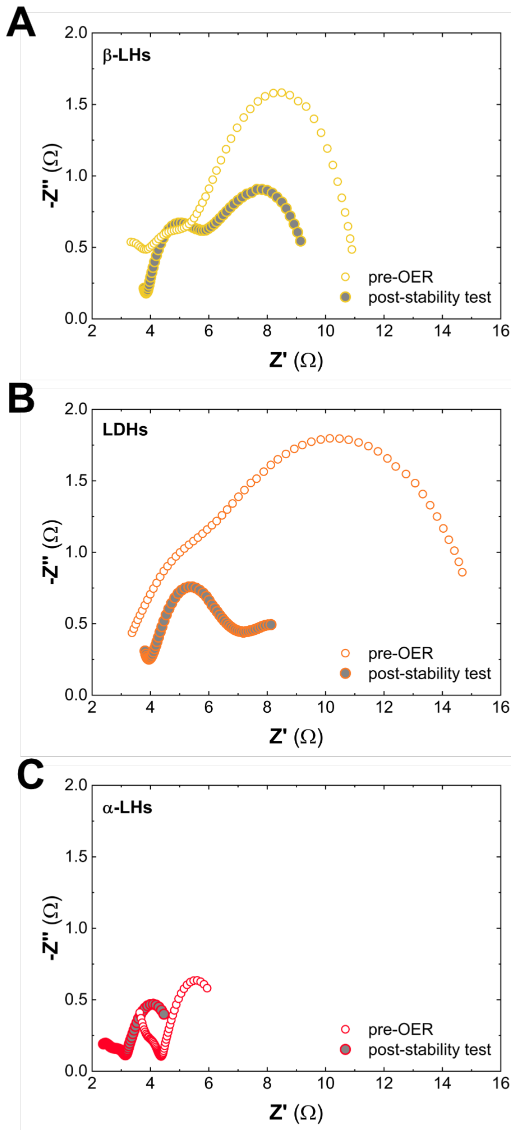

Figure S11. Electrochemical Impedance spectroscopy characterisation before and after the long-term stability tests (see Figure S9) of the different Co-based LH samples deposited on carbon paper and recorded at an overpotential of 0.45 V. β-LH (A), LDH (B) and α-LH (C).

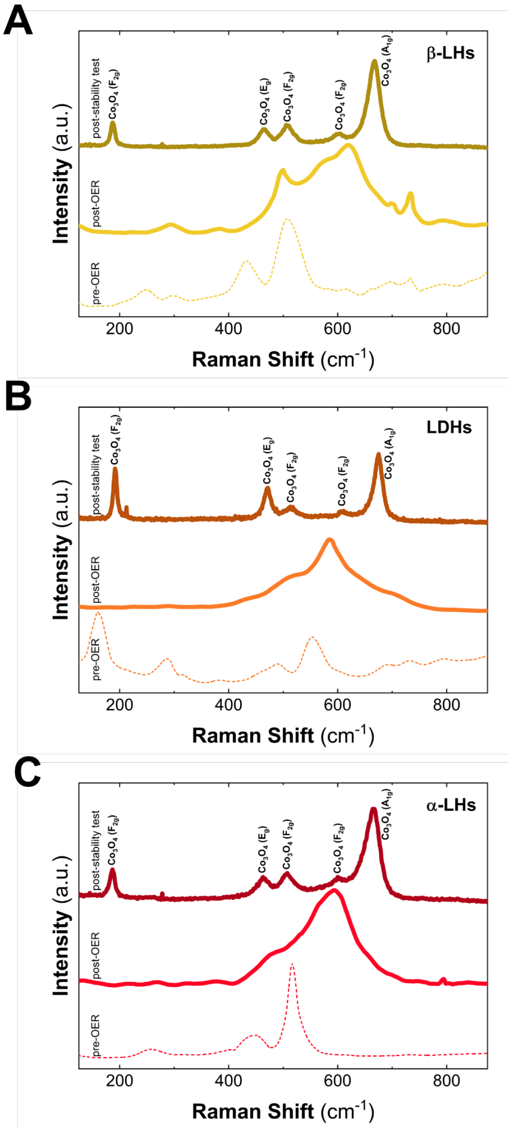

Figure S12. Raman spectroscopy characterization before (pre-OER) and after (post-OER) water oxidation catalysis, and after the long-term ON-OFF stability test (post-stability test) of the different Co-based LH samples performed by chronopotentiometry at 10 mA/cm^2 recorded on a carbon paper electrode collector in 1 M KOH aqueous solution. β-LH (A), LDH (B) and α-LH (C).

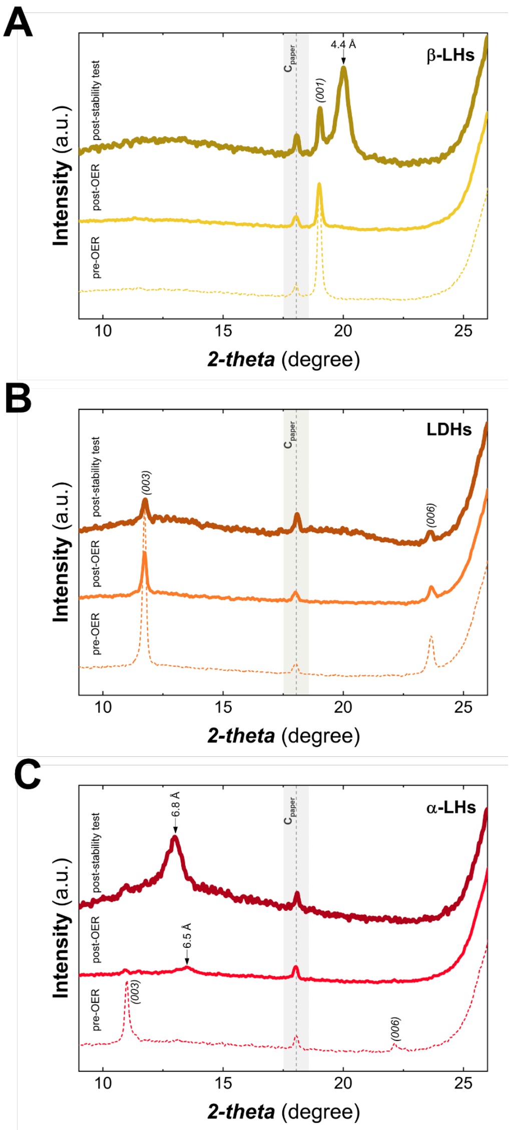

Figure S13. PXRD characterization before (pre-OER) and after (post-OER) water oxidation catalysis, and after the long-term ON-OFF stability test (post-stability test) of the different Co-based LH samples performed by chronopotentiometry at 10 mA/cm^2 recorded on a carbon paper electrode collector in 1 M KOH aqueous solution. β-LH (A), LDH (B) and α-LH (C).

<u>**References**</u>

(1) Liang, J.; Ma, R.; Iyi, N.; Ebina, Y.; Takada, K.; Sasaki, T. Topochemical Synthesis, Anion Exchange, and Exfoliation of Co−Ni Layered Double Hydroxides: A Route to Positively Charged Co−Ni Hydroxide Nanosheets with Tunable Composition. *Chem. Mater.* **2010**, *22* (2), 371–378. https://doi.org/10.1021/cm902787u.

(2) Liu, Z.; Ma, R.; Osada, M.; Iyi, N.; Ebina, Y.; Takada, K.; Sasaki, T. Synthesis, Anion Exchange, and Delamination of Co−Al Layered Double Hydroxide: Assembly of the Exfoliated Nanosheet/Polyanion Composite Films and Magneto-Optical Studies. *J. Am. Chem. Soc.* **2006**, *128* (14), 4872–4880. https://doi.org/10.1021/ja0584471.

(3) Oestreicher, V.; Jobbágy, M. One Pot Synthesis of Mg2Al(OH)6Cl·1.5H2O Layered Double Hydroxides: The Epoxide Route. *Langmuir* **2013**, *29* (39), 12104–12109. https://doi.org/10.1021/la402260m.

(4) Oestreicher, V.; Fábregas, I.; Jobbágy, M. One-Pot Epoxide-Driven Synthesis of M2Al(OH)6Cl·1.5H2O Layered Double Hydroxides: Precipitation Mechanism and Relative Stabilities. *J. Phys. Chem. C* **2014**, *118* (51), 30274–30281. https://doi.org/10.1021/jp510341q.

(5) Arencibia, N.; Oestreicher, V.; A. Viva, F.; Jobbágy, M. Nanotextured Alpha Ni(Ii)–Co(Ii) Hydroxides as Supercapacitive Active Phases. *RSC Advances* **2017**, *7* (10), 5595–5600. https://doi.org/10.1039/C6RA27839F.

(6) Ravel, B.; Newville, M. ATHENA, ARTEMIS, HEPHAESTUS: Data Analysis for X-Ray Absorption Spectroscopy Using IFEFFIT. *J Synchrotron Rad* **2005**, *12* (4), 537–541. https://doi.org/10.1107/S0909049505012719.

(7) Newville, M.; IUCr. *IFEFFIT : interactive XAFS analysis and FEFF fitting*. Journal of Synchrotron Radiation. https://doi.org/10.1107/S0909049500016964.

(8) Rehr, J. J.; Kas, J. J.; Vila, F. D.; Prange, M. P.; Jorissen, K. Parameter-Free Calculations of X-Ray Spectra with FEFF9. *Phys. Chem. Chem. Phys.* **2010**, *12* (21), 5503–5513. https://doi.org/10.1039/B926434E.

(9) Chung, D. Y.; Lopes, P. P.; Farinazzo Bergamo Dias Martins, P.; He, H.; Kawaguchi, T.; Zapol, P.; You, H.; Tripkovic, D.; Strmcnik, D.; Zhu, Y.; Seifert, S.; Lee, S.; Stamenkovic, V. R.; Markovic, N. M. Dynamic Stability of Active Sites in Hydr(Oxy)Oxides for the Oxygen Evolution Reaction. *Nat Energy* **2020**, *5* (3), 222–230. https://doi.org/10.1038/s41560-020-0576-y.

(10) Son, Y. J.; Kawashima, K.; Wygant, B. R.; Lam, C. H.; Burrow, J. N.; Celio, H.; Dolocan, A.; Ekerdt, J. G.; Mullins, C. B. Anodized Nickel Foam for Oxygen Evolution Reaction in Fe-Free and Unpurified Alkaline Electrolytes at High Current Densities. *ACS Nano* **2021**, *15* (2), 3468–3480. https://doi.org/10.1021/acsnano.0c10788.

(11) Giannozzi, P.; Baroni, S.; Bonini, N.; Calandra, M.; Car, R.; Cavazzoni, C.; Ceresoli, D.; Chiarotti, G. L.; Cococcioni, M.; Dabo, I.; Corso, A. D.; Gironcoli, S. de; Fabris, S.; Fratesi, G.; Gebauer, R.; Gerstmann, U.; Gougoussis, C.; Kokalj, A.; Lazzeri, M.; Martin-Samos, L.; Marzari, N.; Mauri, F.; Mazzarello, R.; Paolini, S.; Pasquarello, A.; Paulatto, L.; Sbraccia, C.; Scandolo, S.; Sclauzero, G.; Seitsonen, A. P.; Smogunov, A.; Umari, P.; Wentzcovitch, R. M. QUANTUM ESPRESSO: A Modular and Open-Source Software Project for Quantum Simulations of Materials. *J. Phys.: Condens. Matter* **2009**, *21* (39), 395502. https://doi.org/10.1088/0953-8984/21/39/395502.

(12) Perdew, J. P.; Burke, K.; Ernzerhof, M. Generalized Gradient Approximation Made Simple. *Phys. Rev. Lett.* **1996**, *77* (18), 3865–3868. https://doi.org/10.1103/PhysRevLett.77.3865.

(13) Corso, A. D.; Conte, A. M. Spin-Orbit Coupling with Ultrasoft Pseudopotentials: Application to Au and Pt. *Phys. Rev. B* **2005**, *71* (11), 115106. https://doi.org/10.1103/PhysRevB.71.115106.

(14) Grimme, S. Semiempirical GGA-type density functional constructed with a long-range dispersion correction. *Journal of Computational Chemistry* **2006**, *27* (15), 1787–1799. https://doi.org/10.1002/jcc.20495.

(15) Barone, V.; Casarin, M.; Forrer, D.; Pavone, M.; Sambi, M.; Vittadini, A. Role and

Effective Treatment of Dispersive Forces in Materials: Polyethylene and Graphite Crystals as Test Cases. *Journal of Computational Chemistry* **2009**, *30* (6), 934–939. https://doi.org/10.1002/jcc.21112.
(16) Kokalj, A. XCrySDen—a New Program for Displaying Crystalline Structures and Electron Densities. *Journal of Molecular Graphics and Modelling* **1999**, *17* (3), 176–179. https://doi.org/10.1016/S1093-3263(99)00028-5.
(17) Rossmeisl, J.; Logadottir, A.; Nørskov, J. K. Electrolysis of Water on (Oxidized) Metal Surfaces. *Chemical Physics* **2005**, *319* (1), 178–184. https://doi.org/10.1016/j.chemphys.2005.05.038.
(18) Rossmeisl, J.; Qu, Z.-W.; Zhu, H.; Kroes, G.-J.; Nørskov, J. K. Electrolysis of Water on Oxide Surfaces. *Journal of Electroanalytical Chemistry* **2007**, *607* (1), 83–89. https://doi.org/10.1016/j.jelechem.2006.11.008.
(19) *Electrochemical Methods: Fundamentals and Applications, 2nd Edition* | *Wiley*. Wiley.com. https://www.wiley.com/en-us/Electrochemical+Methods%3A+Fundamentals+and+Applications%2C+2nd+Edition-p-9780471043720 (accessed 2022-11-23).
(20) Kurth, S.; Perdew, J. P.; Blaha, P. Molecular and Solid-State Tests of Density Functional Approximations: LSD, GGAs, and Meta-GGAs. *International Journal of Quantum Chemistry* **1999**, *75* (4–5), 889–909. https://doi.org/10.1002/(SICI)1097-461X(1999)75:4/5<889::AID-QUA54>3.0.CO;2-8.
